# Loss of Neuropeptide Y Signaling Accompanies the Neural-to-Mesenchymal Transcriptional Transition in Glioblastoma: A Multi-Scale Transcriptomic Analysis

**DOI:** 10.3390/ijms27136068

**Published:** 2026-07-06

**Authors:** Fareeha Arshad, Nouran Abualsaud, Arshiya Akbar, Mohammed Imran Khan, Bushra Rasheed, Adnan Hussain, Fahad Ali Alghamdi, Faisal Abdulhameed Farrash, Edwin N. Aroke, Khalid Walid Freij, Itika Arora, Ahmed Yaqinuddin

**Affiliations:** 1 College of Medicine, Alfaisal University, Riyadh 11533, Saudi Arabia; farshad@alfaisal.edu (F.A.); arshiyaakbar2019@gmail.com (A.A.); brasheed01@alfaisal.edu (B.R.); 2 King Abdullah International Medical Research Center, King Saud bin Abdulaziz University for Health Sciences, Riyadh 11426, Saudi Arabia; 3 King Faisal Specialist Hospital and Research Center, Jeddah 23433, Saudi Arabia; mikhan@kfshrc.edu.sa (M.I.K.); hadnan@kfshrc.edu.sa (A.H.); fgalghamdi@kfshrc.edu.sa (F.A.A.); 4 King Faisal Specialist Hospital and Research Center, Riyadh 11211, Saudi Arabia; ffarrash@kfshrc.edu.sa; 5 Department of Acute, Chronic & Continuing Care, School of Nursing, University of Alabama at Birmingham, Birmingham, AL 35294, USA; earoke@uab.edu (E.N.A.); kfreij95@uab.edu (K.W.F.)

**Keywords:** angiogenesis, gene expression, glioblastoma, hypoxia, immune suppression, neuropeptide Y, single-cell RNA sequencing, spatial transcriptomics, transcriptomic analysis, tumor microenvironment

## Abstract

Neuropeptide Y [NPY; encoded by the *NPY* gene] is a widely expressed 36-amino-acid neuropeptide that regulates neuronal function, vascular regulation, and immune regulation; its role in glioblastoma [GBM] remains incompletely characterized. We performed an integrative in silico multi-scale transcriptomic analysis combining bulk RNA-sequencing of IDH-wildtype GBM [*n* = 169] and lower-grade glioma [*n* = 510] surgical resections from TCGA, normal cortical tissue from GTEx [*n* = 207], and four independent GEO validation cohorts of surgical GBM and non-tumor brain specimens [GSE4290, GSE50161, GSE131928 scRNA-seq of ~20,426 cells from 28 patients, and GSE194329 10X Visium spatial transcriptomics from five patients], along with survival modeling, pathway enrichment, single-cell RNA sequencing, spatial transcriptomics, and cell–cell communication analysis. *NPY* and its principal receptor, *NPY1R*, were significantly downregulated in GBM, while genes associated with hypoxia, angiogenesis, invasion, and immune suppression were upregulated. Single-cell analysis showed that *NPY*-axis transcript expression was elevated in neural progenitor-like populations. In contrast, hypoxia and metabolic programs were concentrated in mesenchymal tumors and stromal compartments, indicating distinct cellular contexts. Spatial analysis revealed a weak and heterogeneous relationship between *NPY* and hypoxia signatures, with substantial inter-patient variability and no significant global spatial cross-correlation. These findings indicate that loss of NPY signaling is a consistent feature of GBM and is associated with hypoxia-driven tumor states, while the spatial relationship between *NPY* and hypoxia appears weak, heterogeneous, and patient-specific.

## 1. Introduction

### 1.1. Glioblastoma: Clinical Challenge and the Hypoxic Tumor Microenvironment

Diffuse gliomas are the most common primary malignant brain tumors in adults, with glioblastoma [GBM; World Health Organization, WHO grade 4] representing the most aggressive and lethal subtype. Despite maximal safe surgical resection, concurrent temozolomide chemoradiation, and adjuvant temozolomide, the median overall survival for GBM remains approximately 14–16 months, with fewer than 5% of patients surviving for 5 years [1,2]. The 2021 WHO Classification of Tumors of the Central Nervous System refined the molecular taxonomy of diffuse gliomas, establishing isocitrate dehydrogenase [IDH] mutation status, 1p/19q codeletion, and TERT promoter mutations as the primary determinants of histopathological classification, tumor grading, and overall-survival prognosis in adult diffuse gliomas [1]. Within IDH-wildtype GBM, transcriptomic subtyping has identified proneural, classical, and mesenchymal subtypes, with the mesenchymal subtype exhibiting the most hypoxic, angiogenic, and immunosuppressive transcriptional programs [3,4]. A defining feature of GBM biology is profound intratumoral hypoxia, driven by rapid tumor cell proliferation that outstrips the capacity of an aberrant, poorly organized vasculature to deliver oxygen [5,6]. “Hypoxia-inducible factor [HIF] family transcription factors, principally HIF-1α and HIF-2α [EPAS1], serve as master regulators of this response [5,7,8]. The convergence of these HIF-driven programs with the profoundly immunosuppressive tumor microenvironment [TME] of GBM, characterized by massive infiltration of M2-polarized tumor-associated macrophages [TAMs], regulatory T cells, and myeloid-derived suppressor cells, creates a formidable barrier to effective anti-tumor immunity and therapeutic intervention [9,10].

### 1.2. Neuropeptide Y: Neurobiology and Emerging Roles in Tumor Biology

Neuropeptide Y [NPY], encoded by the *NPY* gene, is among the most abundant neuropeptides in the mammalian CNS, produced primarily by GABAergic inhibitory interneurons and certain populations of neural progenitor cells [11,12]. NPY acts through G protein-coupled receptors, NPY*1R*, NPY*2R*, NPY*4R*, and NPY*5R*, that signal predominantly through G*i/o*-mediated inhibition of adenylyl cyclase, modulation of intracellular calcium flux, and activation of downstream mitogen-activated protein kinase, MAPK, and phosphoinositide 3-kinase, PI3K-Akt signaling cascades [13,14]. In the healthy brain, NPY decreases neuronal firing by activating Gi/o-coupled presynaptic and postsynaptic receptors, suppresses seizure propagation in hippocampal circuits via Y2R-mediated presynaptic inhibition of glutamate release, stimulates acute food intake as the most potent known physiological orexigen through arcuate nucleus–paraventricular nucleus signaling, entrains circadian feeding rhythms through Y5R-dependent hypothalamic circuits, and protects neurons against ischemic and excitotoxic injury through Y1R and Y5R-dependent activation of pro-survival MAPK and PI3K-Akt cascades [13,15].

Beyond the CNS, NPY regulates immune function and tumor biology. In the peripheral immune system, NPY polarizes macrophages to an M2 phenotype, suppresses pro-inflammatory cytokine production, and induces anti-inflammatory polarization via Y1 and Y2 receptor engagement [16,17]. In the tumor context, NPY and its receptors are dysregulated in multiple malignancies, including neuroblastoma, Ewing sarcoma, breast cancer, and hepatocellular carcinoma, in which NPY signaling increases angiogenesis via Y2R-mediated endothelial proliferation, decreases pro-inflammatory macrophage infiltration and cytokine production through Y1R/Y2R engagement, and increases tumor cell survival through receptor-dependent activation of MAPK and PI3K-Akt pro-survival cascades [18,19]. A small body of prior work has examined NPY and NPY receptors in glioma tissue as follows: Körner and colleagues reported NPY-receptor expression in human glioma specimens by autoradiography and immunohistochemistry [20], Gertner et al. described NPY-related signaling in pediatric and adult glial tumors [21], Grouzmann et al. analyzed NPY-receptor distribution in a surgical glioma series [22], and a recent AACR 2024 abstract reported NPY-axis dysregulation in a GBM cohort [23]. These studies were predominantly small, single-cohort, and protein- or autoradiography-based, and reached partially divergent conclusions regarding the directionality of *NPY/NPY*-receptor expression in GBM. The present multi-cohort, multi-platform transcriptomic analysis is designed to reconcile and extend these earlier observations.

Critically, NPY is the most potent known physiological orexigen: episodic NPY secretion from arcuate nucleus neurons into the hypothalamic paraventricular nucleus acutely drives food intake and selectively increases appetite for carbohydrates, with NPY release rising in anticipation of feeding and returning to baseline upon satiety [24,25].

### 1.3. The NPY–Hypoxia Intersection: A Knowledge Gap in GBM Biology

First, HIF-1α has been reported to suppress neuropeptide expression in hypoxic neurons through competitive transcriptional repression and promoter displacement mechanisms, suggesting that the hypoxic GBM microenvironment may directly suppress NPY production [24,25]. Second, NPY receptors, particularly NPY1R, have well-characterized vasoactive properties, mediating vasodilation or vasoconstriction depending on the vessel type and the receptor subtype engaged [13,26]. Given the central role of aberrant vascular biology in GBM, NPY/Y2R signaling, which promotes endothelial cell proliferation, drives angiogenesis, and enhances tumor perfusion in multiple cancer context, represents a biologically compelling but unexplored regulatory circuit in GBM vasculature. Third, the co-expression of NPY receptors with immune checkpoint molecules and immunosuppressive mediators in specific TME cell populations, if confirmed at single-cell resolution, would implicate the NPY axis within the broader immunosuppressive network of GBM [17,27].

Despite these converging lines of evidence, no study to date has provided a comprehensive, multi-omic characterization of the NPY signaling axis in GBM. Prior work has largely focused on individual NPY receptors in isolated experimental systems, without integrating bulk transcriptomic, single-cell, spatial, and network-level analyses to resolve the cell-type specificity, spatial organization, and intercellular communication architecture of NPY signaling in the GBM microenvironment.

### 1.4. Rationale, Study Design, and Aims

To address this knowledge gap, we designed a multi-scale transcriptomic study integrating seven complementary analytical layers to characterize the NPY signaling axis in the context of hypoxia-driven, immunosuppressive GBM. The selection of each module reflects the established biology of the GBM hypoxic and immunosuppressive microenvironment. The NPY-axis genes [*NPY*, *NPY1R*, *NPY2R*, and *NPY5R*] constitute the principal ligand–receptor units active in the human CNS. Hypoxia regulators [*HIF1A* and *EPAS1*] and angiogenesis effectors [VEGFA, ANGPT2, and PDGFRB] were chosen as canonical mediators of HIF-driven vascular remodeling in GBM [5,6,7,8]. Metabolic genes [*LDHA*, *SLC2A1*, *HK2*, and *BNIP3*] capture the Warburg/aerobic glycolysis program characteristic of mesenchymal GBM. The stemness module [*SOX2*, *POU5F1/OCT4*, *NES*, and *PROM1/CD133*] was selected because glioma stem cells [GSCs] reside preferentially in perivascular and hypoxic niches and drive treatment resistance; SOX2 and POU5F1 are master regulators of GSC self-renewal, NES is the prototypical neural-progenitor intermediate filament, and PROM1/CD133 is the most widely validated GSC surface marker [28,29,30]. The invasion module [*MMP2* and *MMP9*] comprises matrix-remodeling proteases that are consistently upregulated in GBM infiltration. The immune-suppression module was deliberately restricted to the following three high-confidence, mechanistically distinct mediators that operate at non-redundant nodes of GBM immune evasion: *CD274 [PD-L1]* as the dominant checkpoint ligand on tumor and myeloid cells, *TGFB1* as the principal cytokine driving regulatory T-cell expansion and M2 macrophage polarization, and *ARG1* as a marker of myeloid-derived suppressor cells and M2-TAM metabolic immunosuppression. Genes with overlapping function or limited expression in the GTEx normal brain reference were excluded to keep the panel compact and statistically tractable for downstream meta-analysis and module scoring.

The analytical pipeline comprises: [i] bulk RNA-seq differential expression analysis of TCGA-GBM [*n* = 169], TCGA-LGG [*n* = 510], and GTEx normal brain specimens using limma-voom [31,32]; [ii] survival modeling using Kaplan–Meier, Cox regression, and least absolute shrinkage and selection operator [LASSO]-penalized Cox approaches [33]; [iii] pathway enrichment analysis using GSEA against MSigDB Hallmarks and over-representation analysis against KEGG, Reactome, and Gene Ontology databases [34,35]; [iv] independent validation and random-effect meta-analysis integrating TCGA with two GEO cohorts [GSE4290 and GSE50161] using the metafor framework [36]; [v] single-cell RNA-sequencing analysis of approximately 20,426 GBM cells from GSE131928 using Seurat v5 with SCTransform, Harmony batch correction, and DoubletFinder [37,38]; [vi] tumor microenvironment characterization including macrophage polarization analysis, CellChat v1.5-based ligand–receptor communication inference [35]; and [vii] 10X Visium spatial transcriptomics from GSE194329 with SPOTlight cell-type deconvolution [28,39], complemented by VIPER-based transcription factor activity inference using DoRothEA regulons and co-expression network analysis [29,30].

The specific aims of this study were: (1) to determine whether NPY-axis genes are differentially expressed in GBM and whether this dysregulation is grade-dependent and subtype-specific; (2) to assess the prognostic significance of NPY-panel gene expression in GBM; (3) to identify the pathway-level programs associated with NPY-axis dysregulation; (4) to validate core findings across independent cohorts through meta-analysis; (5) to resolve the cell-type specificity of NPY-axis and hypoxia program activation at single-cell resolution; (6) to characterize NPY-related intercellular communication in the GBM TME; and (7) to map the spatial organization of NPY and hypoxia programs within GBM tissue architecture.

We report that NPY and its principal receptors are consistently downregulated in GBM across multiple platforms and cohorts, while hypoxia effectors, invasion genes, and immunosuppressive mediators are upregulated. At single-cell resolution, *NPY*-axis activity and HIF-driven hypoxia programs are compartmentalized to distinct cell populations, neural progenitor-like cells and mesenchymal tumor cells, respectively, rather than being co-activated within the same cells. CellChat analysis reveals that *NPY*–*NPY1R*/*NPY2R* ligand–receptor pairs are absent from the intratumoral communication network, and spatial transcriptomics demonstrates a weak negative correlation between NPY and Hypoxia module scores across tumor sections, though with substantial inter-patient variability. These findings reframe the *NPY*–hypoxia relationship in GBM as one of inverse regulation: the progression from normal brain to GBM involves the progressive loss of NPY signaling concomitant with the gain of hypoxia-driven, immunosuppressive transcriptional programs.

## 2. Results

### 2.1. Differential Expression of NPY-Axis Genes Reveals Divergent Regulation in GBM

To characterize the transcriptional landscape of the NPY signaling axis in GBM, we interrogated TCGA data encompassing GBM, LGG, and normal brain specimens. Differential expression analysis of 22 NPY-panel genes across four pairwise comparisons revealed the following striking dichotomy: core NPY-axis genes were downregulated in GBM, whereas hypoxia- and invasion-associated genes were upregulated.

**Figure 1:**
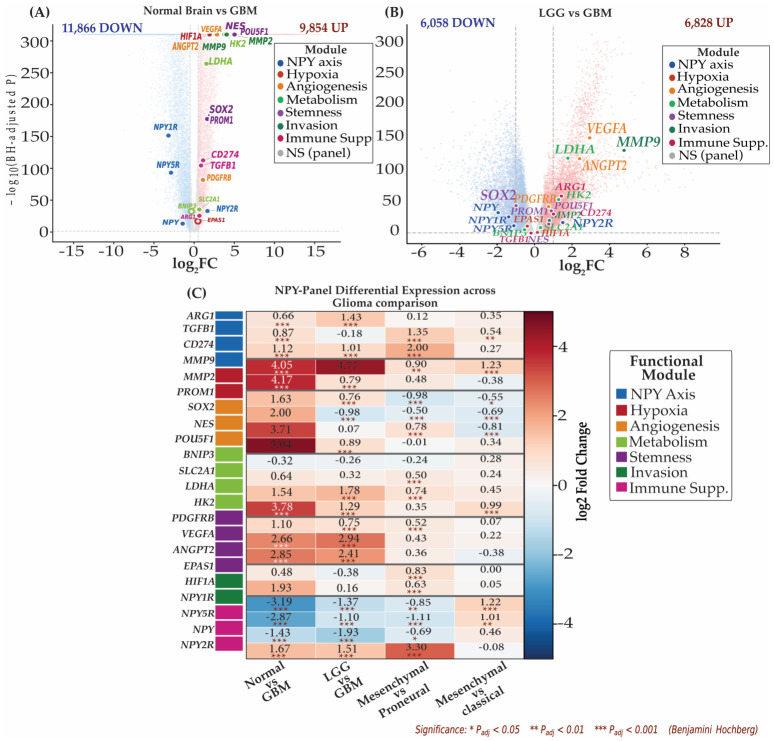
Differential expression of the 22-gene NPY panel in GBM. (**A**) Volcano plot of the normal brain vs. GBM comparison showing log_2_FC vs. −log_10_[BH-adjusted *p*] for 22 NPY-panel genes. Red and blue points indicate significantly upregulated and downregulated genes, respectively [|log_2_FC| > 0.5, BH-adjusted *p* < 0.05]. (**B**) Volcano plot of the LGG vs. GBM comparison demonstrating grade-dependent intensification of fold-changes and (**C**) panel-wide expression heatmap of all 22 *NPY*-panel genes across the TCGA GBMLGG cohort [*n* = 702], with unsupervised hierarchical clustering. See Supplementary Figure S1a,b for subtype-stratified volcano plots. Supplementary Tables S1–S4 provide full DEA results.

In the normal brain vs. GBM comparison, the core *NPY* ligand and receptors *NPY*, *NPY1R*, and *NPY5R* were significantly downregulated, whereas *HIF1A*, *VEGFA*, *MMP2*, *MMP9*, *HK2*, and *POU5F1* were significantly upregulated [Figure 1A]. *NPY2R* was the sole *NPY* receptor upregulated, indicating receptor-specific dysregulation, while *EPAS1* and *BNIP3* showed sub-threshold changes. Full log_2_FC values, adjusted *p*-values, and direction calls for all 22 panel genes are provided in Table 1 and Supplementary Table S1.

The LGG vs. GBM comparison confirmed the following grade-dependent intensification: *VEGFA* [+2.94], MMP9 [+4.77], LDHA [+1.78], and *ANGPT2* [+2.41] showed larger fold changes than in the normal vs. GBM comparison [Figure 1B]. Subtype-stratified analyses demonstrated that mesenchymal GBM showed significantly elevated hypoxia-module and immune-exclusion signatures relative to proneural and classical subtypes [Supplementary Figure S1a,b]. A panel-wide expression heatmap confirmed the consistent inverse correlation between NPY-axis genes and hypoxia/invasion module genes across the TCGA cohort [Figure 1C; Supplementary Tables S1–S4].

### 2.2. Prognostic Significance of NPY-Panel Genes in GBM

**Figure 2:**
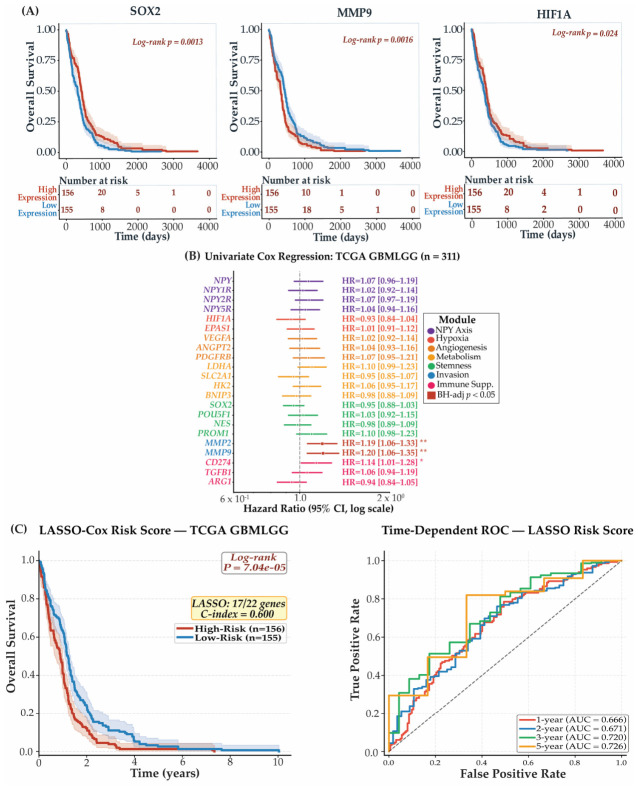
Prognostic significance of NPY-panel genes in the TCGA GBMLGG cohort [*n* = 311]. (**A**) Kaplan–Meier survival curves for selected NPY-panel genes stratified by median expression [high vs. low]. Log-rank *p*-values are shown; multiple-testing correction was performed using the BH method [α = 0.05]. (**B**) Forest plot of univariate Cox proportional hazards regression for all 22 panel genes, displaying hazard ratios with 95% confidence intervals. Expression values were z-score-standardized before model fitting where * = *p* < 0.05; and ** = *p* < 0.01 (BH-adjusted). (**C**) LASSO-penalized Cox regression risk-score stratification: patients dichotomized into high- and low-risk groups by the median composite risk score, with Kaplan–Meier curves and time-dependent receiver operating characteristic [ROC analysis at 1-, 2-, 3-, and 5-year endpoints].

Kaplan–Meier analysis demonstrated that high expression of hypoxia and angiogenesis genes [*HIF1A*, *VEGFA*, and *ANGPT2*] and invasion genes [*MMP2*, *MMP9*] was associated with reduced overall survival in the TCGA GBMLGG cohort, whereas elevated *NPY* and *NPY1R* expression was associated with longer overall survival [Figure 2A]. Univariate Cox proportional hazards regression confirmed these associations at the gene level, with forest plot visualization summarizing hazard ratios and confidence intervals for all 22 panel genes [Figure 2B; Table 2].

A LASSO-penalized Cox regression model selected a parsimonious multi-gene signature from the 22-gene panel and stratified patients into high- and low-risk groups with significantly divergent survival trajectories [Figure 2C]. Time-dependent ROC analysis at 1-, 2-, 3-, and 5-year endpoints confirmed discriminatory accuracy [area under the curve, AUC = 0.666, 0.671, 0.720, and 0.726, respectively].

### 2.3. Pathway Enrichment Identifies Hypoxia, EMT, and Immune Signaling as Central Programs

**Figure 3:**
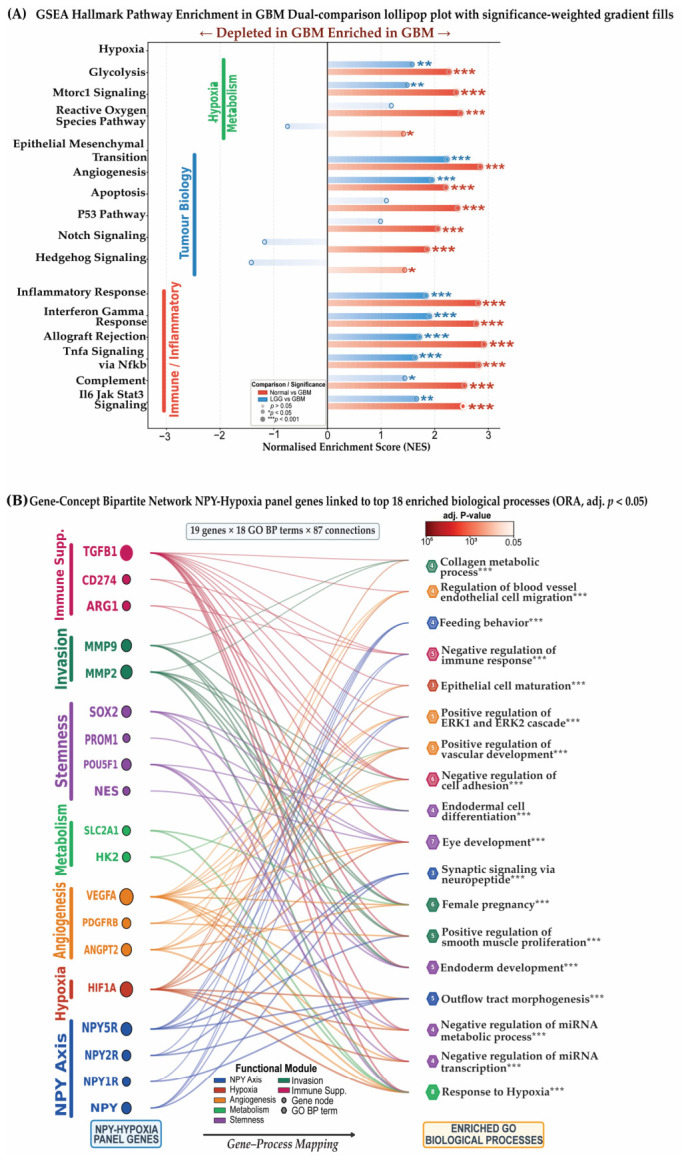
Pathway enrichment analysis of the **NPY**-hypoxia gene panel in GBM. (**A**) GSEA of Hallmark pathways comparing normal vs. GBM [red] and LGG vs. GBM [blue] contrasts. Bars represent normalized enrichment scores [NESs]; bar opacity reflects adjusted *p*-value significance. Pathways are grouped by functional category, indicated by colored ribbons on the left margin. Asterisks denote statistical significance [* *p* < 0.05, ** *p* < 0.01, and *** *p* < 0.001; BH correction] and (**B**) gene-concept bipartite network depicting relationships between NPY-panel genes and enriched GO Biological Process terms. Nodes are color-coded by functional module [see legend in figure]. Outer nodes represent enriched GO BP terms [adjusted *p* < 0.05], sized by significance. Edges connect genes to their annotated biological processes.

GSEA using the MSigDB Hallmarks collection [normal vs. GBM] identified broad pathway activation. The most significantly enriched programs included Allograft Rejection [NES = 2.92], G2M Checkpoint [NES = 3.11], E2F Targets [NES = 3.07], Epithelial-to-Mesenchymal Transition [EMT; NES = 2.85], Inflammatory Response [NES = 2.81], IL-6/JAK/STAT3 Signaling [NES = 2.53], Complement [NES = 2.55], Glycolysis [NES = 2.40], and Hypoxia [NES = 2.27], all with adjusted *p* < 2.5 × 10^−10^ [Figure 3A; Supplementary Figure S2; Supplementary Table S5]. LGG vs. GBM GSEA confirmed similar patterns with EMT [NES = 2.23], Angiogenesis [NES = 1.95], and Hypoxia [NES = 1.58] among the top enrichments [Supplementary Table S6].

Over-representation analysis of significant NPY-panel DEGs against KEGG identified HIF-1 signaling pathway [six genes; fold enrichment = 22.4, *p =* 1.61 × 10^−7^] and Central Carbon Metabolism in Cancer [five genes; fold enrichment = 28.8] as the most enriched cascades [Figure 3B; Supplementary Table S7]. GO Biological Process analysis identified Response to Hypoxia [8/20 genes; fold enrichment = 20.95, *p =* 1.66 × 10^−9^] and Synaptic Signaling via Neuropeptide [3/20 genes, including NPY, NPY2R, NPY5R; fold enrichment = 231.0] [Supplementary Tables S8 and S9] as top terms.

### 2.4. Cross-Cohort Meta-Analysis Validates Core Dysregulation but Reveals Receptor Heterogeneity

**Figure 4:**
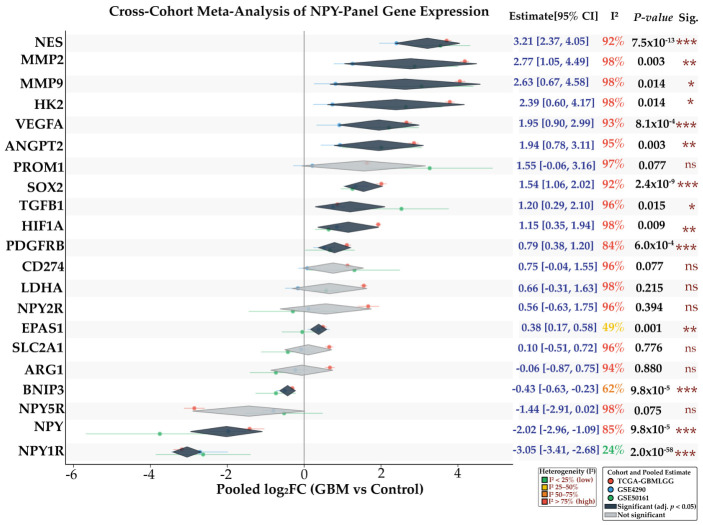
Cross-cohort meta-analysis of NPY-panel gene expression. Random-effect meta-analytic forest plot of pooled log_2_FC estimates for all 22 NPY-panel genes across three independent cohorts [TCGA-GBMLGG, GSE4290, and GSE50161]. Diamonds represent pooled effect sizes with 95% confidence intervals. Individual cohort estimates are shown as colored points [TCGA, red; GSE4290, blue; and GSE50161, green]. Significance: * *p* < 0.05, ** *p* < 0.01, and *** *p* < 0.001 [BH correction]. *I*^2^ heterogeneity statistics are annotated for each gene.

A random-effect meta-analysis integrating three independent cohorts [TCGA-GBMLGG, GSE4290, and GSE50161] confirmed the robustness of the core findings while revealing substantial heterogeneity among NPY receptor genes. *NPY* downregulation was conserved across all three cohorts [pooled estimate = −2.02, *p* = 2.22 × 10^−5^, and I^2^ = 84.8%] [Figure 4]. *NPY1R* was the most validated gene, with consistent downregulation across cohorts [pooled estimate = −3.05, *p* = 9.64 × 10^−60^, and I^2^ = 24.0%, indicating low heterogeneity] [Table 3].

Among upregulated genes, *VEGFA* [+1.95, *p* = 2.71 × 10^−4^], *ANGPT2* [+1.94, *p* = 1.07 × 10^−3^], *MMP2* [+2.77, *p* = 1.64 × 10^−3^], and *HIF1A* [+1.15, *p* = 4.58 × 10^−3^] were significantly upregulated in the meta-analysis, although all exhibited high between-study heterogeneity [I^2^ > 84%]. Critically, *NPY2R* failed to replicate across GEO cohorts. Despite upregulation in TCGA [logFC = +1.67], GSE4290 showed only a marginal non-significant change [+0.11, adj. *p* = 0.58] and GSE50161 showed a non-significant reversal [−0.30, adj. *p* = 0.69]. The meta-analytic estimate was non-significant [pooled estimate = +0.56, *p* = 0.356, and I^2^ = 96.5%] [Figure 4]. Similarly, NPY5R trended towards downregulation but did not reach significance [pooled estimate = −1.44, *p* = 0.053, and I^2^ = 97.9%]. These findings indicate that NPY2R upregulation may be TCGA-specific and should be interpreted with caution [Supplementary Figure S3; Supplementary Table S10]. Among NPY receptors, only NPY*1R* showed robust low-heterogeneity downregulation across cohorts, whereas NPY*2R* and NPY*5R* showed substantial between-study heterogeneity and did not replicate consistently.

### 2.5. Single-Cell Transcriptomics Reveals Cell-Type Compartmentalization of NPY and Hypoxia Programs

**Figure 5:**
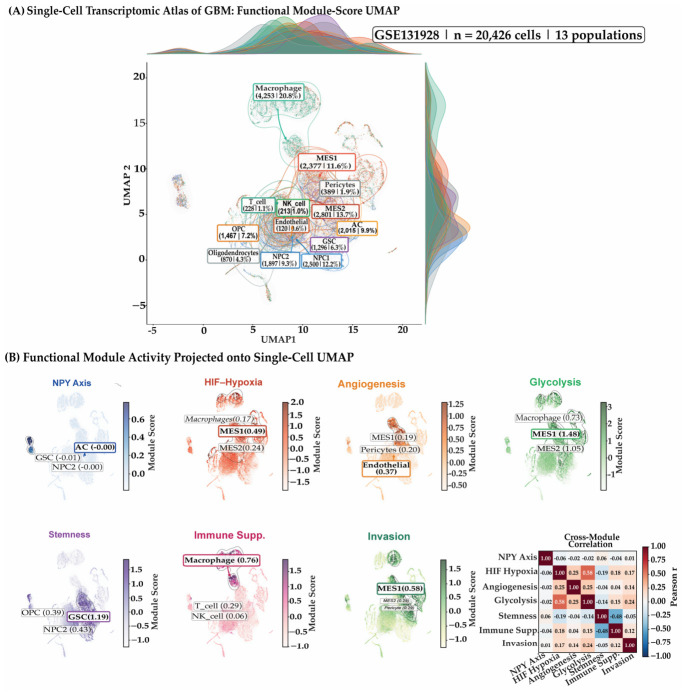
Single-cell transcriptomic landscape of NPY-Hypoxia modules in GBM. (**A**) Uniform manifold approximation and projection [UMAP] of ~20,426 GBM cells [GSE131928] annotated into 13 cell populations and (**B**) module score distributions across cell types for the seven NPY-Hypoxia functional modules. The NPY axis module is highest in neural-progenitor-like populations [*NPC1*, *NPC2*, and *OPC*], while HIF-Hypoxia and Glycolysis modules are highest in MES1 and MES2 subtypes.

Analysis of 20,426 GBM cells from GSE131928 resolved 13 major cell populations: six tumor subtypes [MES1, MES2, AC, OPC, NPC1, and NPC2], glioma stem cells [GSCs], macrophages, T cells, NK cells, endothelial cells, pericytes, and oligodendrocytes [Figure 5A; Supplementary Table S11]. Module score analysis revealed a critical finding: the NPY_Axis and HIF_Hypoxia modules were not co-activated; instead, they showed cell-type-specific segregation. The NPY_Axis module scored highest in NPC2 [+0.012], NPC1 [+0.008], OPC [+0.012], and GSC [+0.003] populations, consistent with NPY’s known neural-progenitor origin. In contrast, the HIF_Hypoxia module was highest in NK cells [+0.145], MES1 [+0.149], Pericytes [+0.111], and MES2 [+0.071], while it was strongly negative in NPC1 [−0.226], OPC [−0.118], and oligodendrocytes [−0.249]. The Glycolysis module was most activated in MES1 cells [+0.637], consistent with Warburg metabolism in mesenchymal GBM [Figure 5B; Table 4; Supplementary Table S12].

The immune suppression module [*CD274*, *TGFB1*, *ARG1*, *IDO1*, and *HAVCR2*] was strongly activated in macrophages [+0.211] and T cells [+0.105], while being negative across all tumor subtypes. This indicates that immunosuppressive signaling occurs predominantly within the immune infiltrate rather than directly in tumor cells [Supplementary Figure S4].

### 2.6. Tumor Microenvironment: Macrophage Polarization and Intercellular Communication

**Figure 6:**
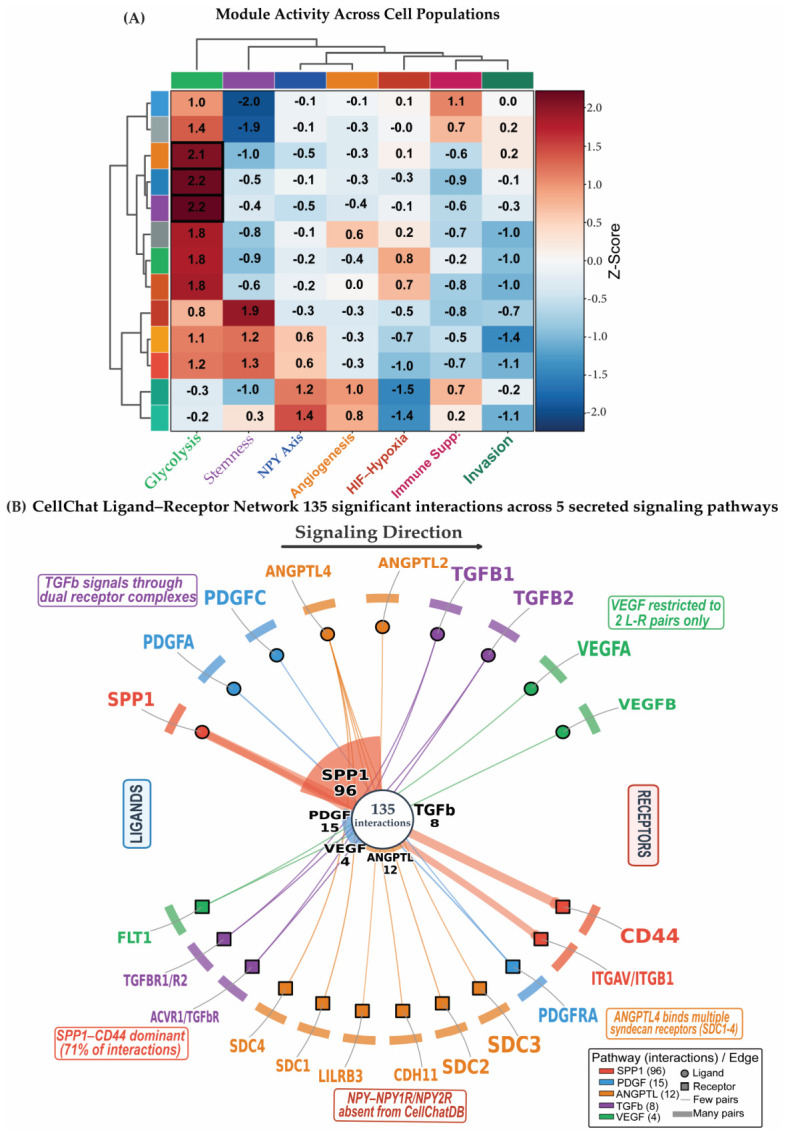
Tumor microenvironment characterization and intercellular communication. (**A**) Heatmap of mean module scores for seven functional programs across 13 cell populations, with hierarchical clustering of both axes and (**B**) CellChat circle plot of significant ligand–receptor interaction counts between cell types, filtered for NPY-relevant pathways [*SPP1*, *PDGF*, *ANGPTL*, *TGFb*, and *VEGF*].

Macrophage polarization analysis demonstrated heterogeneous M1/M2 scoring across TAM subpopulations, with the NPY-axis module showing variable expression within the macrophage compartment [Figure 6B; Supplementary Figure S5; Supplementary Table S13]. Module × cell-type heatmap analysis confirmed that the immune suppression module was most strongly activated in macrophages and T cells, while the angiogenic module peaked in endothelial cells [+0.066] and MES1 tumor cells [+0.058] [Figure 6A]. CellChat analysis identified 1842 significant ligand–receptor interactions across the 13 cell types [Supplementary Table S14]. When filtered for pathways relevant to the NPY-hypoxia-immune axis, 135 interactions were retained, comprising: *SPP1* signaling [96 interactions, 71.1%], *PDGF* [15, 11.1%], *ANGPTL* [12, 8.9%], *TGFb* [eight, 5.9%], and *VEGF* [four, 3.0%] [Figure 6B; Supplementary Table S15]. Circle and bubble plot analyses revealed that tumor cells [particularly MES1/MES2] and macrophages were the dominant senders and receivers of these signals [Figure 6B; Supplementary Tables S16 and S17].

Importantly, *NPY*–*NPY1R/NPY2R* ligand–receptor pairs are not represented in the CellChatDB database, precluding direct inference of NPY-mediated intercellular communication from this analysis. The absence of direct NPY signaling interactions, combined with the low expression of NPY1R across all cell types [<2% of cells in any population], suggests that NPY’s role in the GBM microenvironment is primarily that of a lost neuroprotective signal rather than an active intercellular communication pathway. The *SPP1* [osteopontin] signaling axis is a dominant macrophage–tumor communication pathway in this dataset.

To independently verify this finding, we implemented a permutation-based ligand–receptor interaction test that directly evaluated NPY–NPY1R, NPY–NPY2R, and NPY–NP*Y5R* communication across all 13 cell-type pairs. No NPY L-R pair passed a 5% expression threshold in either the sender or receiver population: *NPY* was expressed in at most 7.8% of cells [Tumor_NPC2], while NPY*1R* peaked at 2.0% [pericytes], NPY*2R* at 2.5% [Tumor_AC], and NPY*5R* at 1.5% [pericytes]. Even at a relaxed 1% threshold, communication probabilities for all NPY pairs were four orders of magnitude below those of validated control pairs [*VEGFA*–FLT1; *p* ~ 10^−5^ *vs.* 10^−1^]. By contrast, *VEGFA*–*FLT1* signaling between MES1 tumor cells and endothelial cells showed robust communication [35.0% and 39.5% expression, respectively]. This indicates that NPY-mediated intercellular signaling is not detectable at biologically meaningful transcript levels in this dataset [Supplementary Table S18].

### 2.7. Spatial Transcriptomics Maps NPY and Hypoxia to Weakly Inversely Correlated Tumor Zones

**Figure 7:**
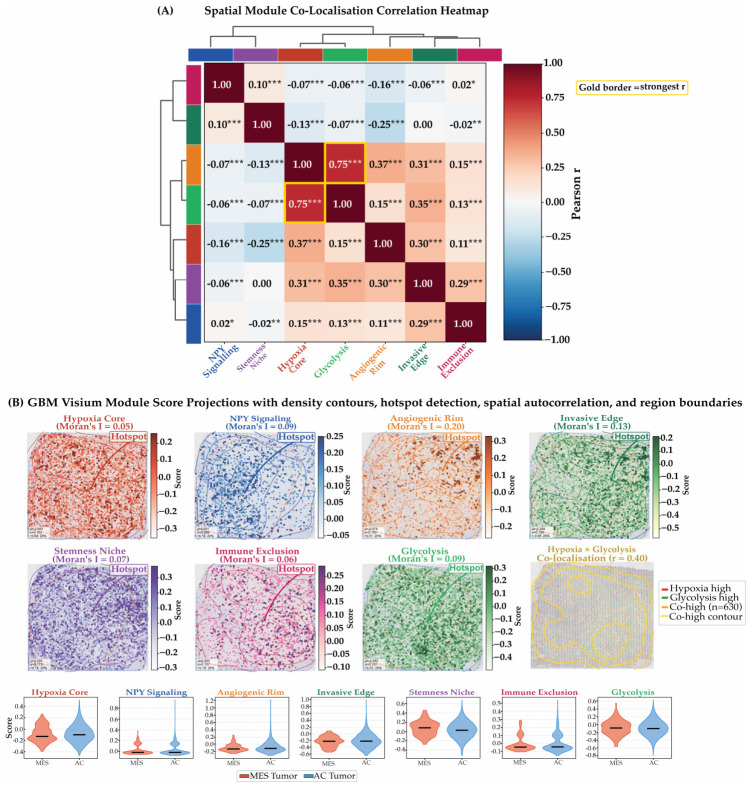
Spatial transcriptomic architecture of ***NPY***-Hypoxia modules in GBM. (**A**) Spatial module co-localization heatmap showing Pearson correlation coefficients between seven *NPY*-Hypoxia module scores across 16,032 Visium spots from five GBM specimens [GSE194329]. Hierarchical clustering [Ward’s linkage] with significance annotations [* *p* < 0.05, ** *p* < 0.01, and *** *p* < 0.001] and (**B**) module score projections onto a representative GBM tissue section. Each panel displays one module’s activity overlaid on the hematoxylin and eosin [H and E] image, with density contours delineating spatial hotspots. The eighth panel presents a Hypoxia Core × Glycolysis dual-color overlay. Bottom strip: split-violin plots of module score distributions per spatial region.

Spatial gene expression analysis of six 10X Visium sections from five GBM patients [GSE194329] resolved discrete intratumoral regions through module-score-based annotation [Figure 7A; Supplementary Figures S6 and S7]. Spatial projection of all seven module scores revealed that the Hypoxia Core and Glycolysis modules were strongly co-localized [Pearson r = 0.746 across all spots], concentrated within tumor core and pseudo-palisading regions. The Invasive Edge module co-localized with both Hypoxia Core [r = 0.312] and Angiogenic Rim [r = 0.295] [Figure 7A]. To move beyond pairwise Pearson correlations, which ignore spatial structure, we computed bivariate Moran’s I [k = six nearest neighbors, 999 permutations] between NPY Signaling and Hypoxia Core module scores. The pooled statistic across all 16,032 spots was I_biv = 0.002 [*p* = 0.359], indicating no global spatial cross-correlation. Per-sample analysis revealed the following patient-level heterogeneity: GBM5_1 showed a significant negative spatial association [I_biv = −0.124, *p* = 0.001]; GBM4 showed a significant positive association [I_biv = +0.113, *p* = 0.001]; and the remaining samples were non-significant [Figure 7A]. As a positive control, Hypoxia Core × Glycolysis yielded I_biv = 0.295 [*p* = 0.001], confirming the method’s sensitivity. These results indicate that the NPY–hypoxia spatial relationship is patient-specific rather than a universal anti-correlation [Figure 7B]. SPOTlight cell-type deconvolution confirmed spatial segregation of cell-type composition across tumor regions, with macrophage-enriched zones preferentially localizing to hypoxic core areas, consistent with hypoxia-driven myeloid recruitment [Figure 7B; Supplementary Figure S8].

### 2.8. Transcription Factor Activity and Co-Expression Network Analysis Identifies Regulatory Hubs

**Figure 8:**
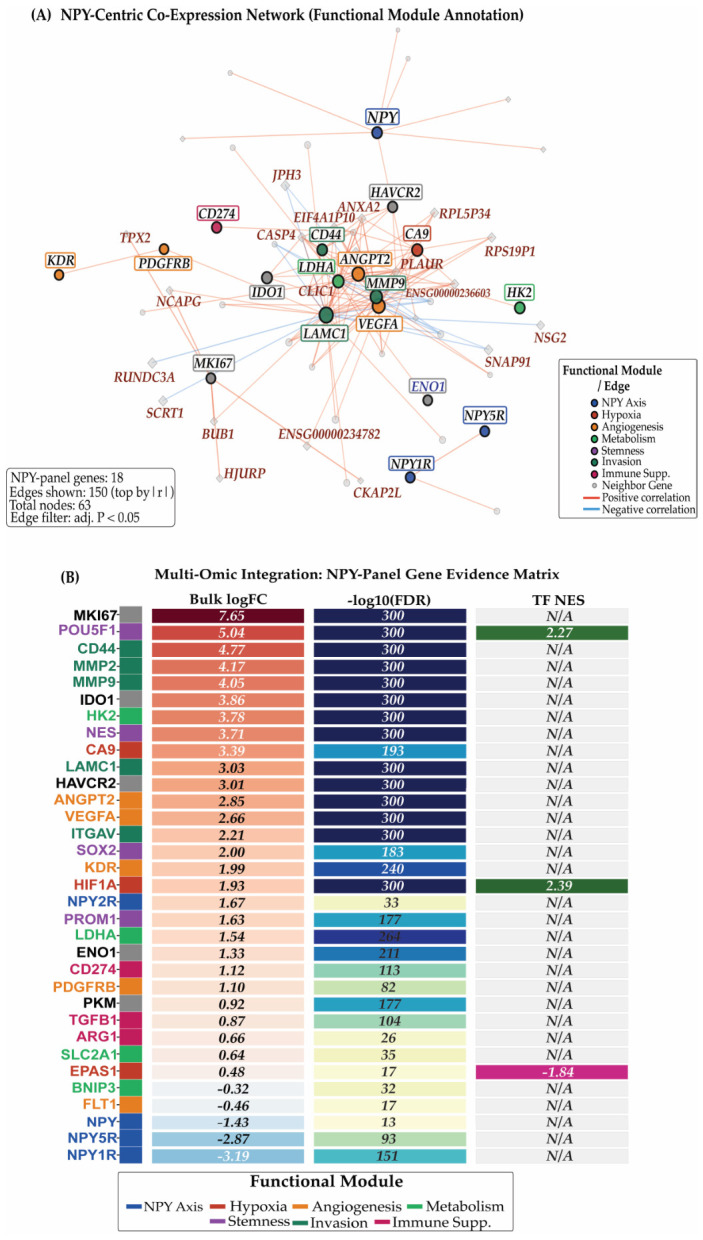
Multi-omic integration of the NPY-Hypoxia signaling axis in GBM. (**A**) NPY-centric co-expression network from the TCGA-GBM cohort [adj. *p* < 0.05]. Nodes are colored by functional module assignment. Gray nodes represent co-expressed neighbor genes. Red and blue edges denote positive and negative correlations, respectively. (**B**) Multi-omic gene evidence matrix for 30 NPY-panel and associated genes. Horizontal bars display bulk RNA-seq log_2_FC [normal vs. GBM], −log_10_[false discovery rate, FDR], and VIPER-inferred transcription factor NES. Gene names are colored by functional module. N/A indicates genes without TF activity inference. Gene label colors indicate functional module; ‘Bulk logFC’ uses a blue-to-red scale (down- to upregulated); ‘−log10(FDR)’ uses a white-to-dark-blue scale (low-to-high significance); ‘TF NES’ uses green (positive) to magenta (negative) to indicate transcription factor activity direction.

VIPER-based transcription factor activity inference identified *HIF1A*, *EPAS1*, *STAT3*, *RELA [NF-κB]*, and *SP1* among the 15 tracked NPY-axis-relevant TFs with significantly elevated activity in the GBM cohort [Figure 8A]. The identification of STAT3 is consistent with its known role as a HIF-1α transcriptional co-activator and a downstream effector of IL-6/JAK signaling, corroborating the GSEA enrichment of the IL-6/JAK/STAT3 Hallmark set [NES = 2.53]. Grade-stratified analysis showed a progressive increase in HIF-family TF activity from LGG to GBM [Supplementary Figure S9A].

The co-expression network, constructed from the top co-expressed gene pairs [|r| ≥ 0.50, adj. *p* < 0.05], resolved clear community structure with a hypoxia-angiogenesis cluster [*VEGFA–ANGPT2–MMP9–LDHA–HK2*] separated from NPY-associated nodes in the network layout [Figure 8A]. Louvain community detection confirmed that NPY and NPY1R occupy a neural-identity community distinct from the tumor-associated hypoxia/invasion community, providing network-level confirmation of the cell-type compartmentalization observed in the single-cell analysis.

An integrative multi-omic heatmap synthesizing bulk log^2^FC, meta-analytic significance, and VIPER TF activity for all 22 NPY-panel genes confirmed the convergent pattern as follows: upregulated genes [MMP2, MMP9, HK2, VEGFA, POU5F1, and NES] clustered together in tumor-associated network communities, while NPY, NPY1R, and NPY5R formed a separate cluster characterized by downregulation in GBM and assignment to neural-identity network communities [Figure 8B].

## 3. Discussion

### 3.1. Overview: Loss of NPY Signaling as a Feature of Hypoxic, Immunosuppressive GBM

The present study provides a comprehensive multi-omic transcriptomic analysis of the NPY signaling axis in GBM, integrating bulk transcriptomics from TCGA and two independent GEO validation cohorts, single-cell RNA sequencing from approximately 20,426 GBM cells, 10X Visium spatial transcriptomics, CellChat-based inference of intercellular communication, and VIPER transcription factor activity profiling. The central finding is that NPY and its principal receptor, NPY1R, are consistently downregulated in GBM across the TCGA cohort and two independent GEO validation cohorts, while hypoxia effectors, invasion genes, and immunosuppressive mediators are upregulated. We note that this observation is not uniformly concordant with the existing literature: earlier studies have variously reported retained or even elevated NPY/NPY-receptor immunoreactivity in subsets of glioma tissue [20,21,22,23]. Several factors plausibly reconcile these discrepancies as follows: [i] prior studies were predominantly small immunohistochemical or single-cohort analyses, often pooling IDH-mutant and IDH-wildtype tumors or including lower-grade gliomas, whereas the present meta-analysis is restricted to IDH-wildtype GBM and integrates > 700 transcriptomes across three independent platforms; [ii] protein-level immunostaining detects both tumor-cell and entrapped neuronal/peritumoral NPY, which can mask the GBM-intrinsic transcriptional loss reported here; and [iii] receptor-specific findings differ across studies in part because *NPY2R* and *NPY5R* show high cross-platform heterogeneity in our meta-analysis [I^2^ > 96%], consistent with reported assay-dependent variability. We therefore present the current finding as transcriptomically robust for NPY, while explicitly acknowledging that protein-level validation in IDH-stratified, primary-vs-recurrent GBM cohorts is required to fully reconcile our results with prior immunohistochemical reports. At the single-cell level, NPY-axis activity and HIF-driven hypoxia programs are compartmentalized to distinct cell populations, neural progenitor-like cells and mesenchymal tumor cells, respectively, rather than being co-activated within the same cells. This reframes the NPY–hypoxia relationship as one of inverse regulation as follows: the progression from normal brain to GBM involves the loss of neuropeptide Y signaling concomitant with the gain of hypoxia-driven, immunosuppressive transcriptional programs.

### 3.2. Robust Downregulation of NPY and NPY1R with Grade-Dependent Intensification

The TCGA differential expression analysis demonstrated that *NPY* [−1.43 log^2^FC] and *NPY1R* [−3.19 log^2^FC] are among the most strongly suppressed genes in the NPY panel in GBM relative to normal brain. This finding was independently validated by meta-analysis as follows: *NPY1R* showed the most robust cross-cohort consistency [pooled estimate = −3.05, I^2^ = 24.0%], with *NPY* also consistently downregulated [pooled estimate = −2.02, I^2^ = 84.8%]. The low heterogeneity for *NPY1R* is particularly noteworthy, as it indicates that this receptor’s suppression is a conserved feature of GBM biology rather than a platform-specific or cohort-specific artifact [40]. The contrasting behavior of *NPY2R* upregulated in TCGA [+1.67 log^2^FC] but non-significant in both GEO cohorts [meta *p* = 0.356, I^2^ = 96.5%]—warrants careful interpretation. *NPY2R*’s TCGA expression may reflect cell-type composition effects that are not conserved across microarray platforms, given its low baseline expression and cell-type specificity observed in our single-cell data. The failure to validate across cohorts means that *NPY2R* should not be considered a reliable GBM biomarker without further protein-level validation [41]. A key concern in bulk transcriptomic analyses of GBM is that observed differential expression may reflect changes in cell-type composition—particularly the loss of neural lineage cells, rather than gene-intrinsic regulatory changes. To address this, we performed analytical composition correction using scRNA-seq-derived cell-type expression profiles and estimated neural lineage fractions [normal brain ~ 65%, GBM ~ 30.7%]. For *NPY1R*, the composition-expected log_2_FC based on neuron loss alone was −0.05, yet the observed value was −3.19, leaving 98.5% of the downregulation unexplained by composition shift [residual log_2_FC = −3.14]. This strongly supports a gene-intrinsic regulatory mechanism for *NPY1R* suppression in GBM—consistent with potential promoter hypermethylation or HIF-mediated transcriptional repression. For NPY, the composition-expected shift was −0.84 [reflecting its neural cell enrichment], with 41% of the observed downregulation [residual log_2_FC = −0.58] remaining after correction. The substantial residual for both genes indicates that their suppression in GBM cannot be attributed solely to the loss of NPY-expressing neural populations and implicates active transcriptional silencing mechanisms.

### 3.3. NPY and Hypoxia Programs Are Compartmentalized, Not Co-Activated

Perhaps the most informative finding of this study is the single-cell resolution of NPY-axis and HIF-hypoxia programs to distinct cellular compartments. The NPY_Axis module scored highest in NPC1, NPC2, OPC, and GSC populations, cells with neural progenitor identity, while HIF_Hypoxia was concentrated in MES1 tumor cells, pericytes, and NK cells. The Glycolysis module, which shares effectors with the HIF response [LDHA, HK2], was most activated in MES1 cells [+0.637], confirming the Warburg phenotype of mesenchymal GBM. This compartmentalization is biologically coherent as follows: *NPY* is a neuropeptide produced by inhibitory interneurons and neural progenitors, while HIF-driven transcriptional programs are activated in cells exposed to oxygen deprivation in the necrotic tumor core [42,43].

The spatial transcriptomics data provide concordant, though nuanced, support. Bivariate Moran’s I analysis, which, unlike Pearson correlation, accounts for the spatial autocorrelation structure inherent to Visium data yielded a pooled I_biv of 0.002 [*p* = 0.359] between Hypoxia Core and NPY Signaling module scores, indicating no systematic spatial cross-correlation at the global level. However, per-sample analysis revealed significant patient-level heterogeneity as follows: GBM5_1 exhibited significant negative spatial association [I_biv = −0.124, *p* = 0.001], while GBM4 showed significant positive association [I_biv = +0.113, *p* = 0.001], with the remaining four samples non-significant. This heterogeneity suggests that the NPY–hypoxia spatial relationship depends on the preservation of neural progenitor populations in the peritumoral microenvironment and is not a universal feature of GBM [6,28]. As a positive control, Hypoxia Core × Glycolysis yielded I_biv = 0.295 [*p* = 0.001], confirming the expected metabolic co-localization and validating the spatial method’s sensitivity. The weak and heterogeneous NPY spatial signal is consistent with the low abundance of NPY-producing cells in the resected tissue sections.

### 3.4. The Immunosuppressive TME Operates Through SPP1, Not NPY, Signaling

A critical finding that distinguishes the present analysis from our initial hypothesis is the absence of *NPY–NPY1R/NPY2R* interactions in the CellChat output. The NPY ligand–receptor pairs are not represented in the CellChatDB database, and even beyond its coverage, NPY1R was expressed in fewer than 2% of cells across all 13 annotated populations, indicating minimal receptor availability for intercellular signaling. The 135 “NPY-relevant” interactions identified through pathway filtering were instead dominated by SPP1/osteopontin signaling [96 interactions, 71.1%], with PDGF [11.1%], ANGPTL [8.9%], TGFb [5.9%], and VEGF [3.0%] comprising the remainder [28].

To independently verify this finding beyond CellChat’s database limitations, we performed a custom permutation-based ligand–receptor interaction test for all three NPY pairs [NPY–NPY1R, NPY–NPY2R, and NPY–NPY5R] across all 13 × 13 cell-type sender–receiver combinations. None of the 507 tested pairs passed the minimum 5% co-expression threshold required for biologically meaningful signaling: NPY reached a maximum of 7.8% expression in NPC2, while NPY1R peaked at 2.0% in pericytes, and no sender–receiver pair achieved simultaneous ligand and receptor expression above 5%. By contrast, the positive control pair VEGFA–FLT1 showed 35–40% expression in macrophage and pericyte populations with robust communication scores. This database-independent analysis confirms that NPY ligand–receptor signaling is not merely absent from CellChat’s curated pathways but is biologically inactive at the transcript level in the GBM microenvironment.

This result shifts the mechanistic narrative as follows: rather than NPY actively driving immunosuppressive intercellular communication, the loss of NPY signaling in GBM coincides with the dominance of SPP1-mediated macrophage–tumor crosstalk. SPP1 has been independently identified as a key mediator of tumor-associated macrophage recruitment and immunosuppression in GBM [9,27]. The co-presence of TGFB and VEGF interactions, alongside SPP1, reflects the canonical immunosuppressive communication network of the GBM TME, which operates independently of neuropeptide signaling.

### 3.5. Co-Expression Network and TF Activity Confirm NPY–Hypoxia Module Segregation

The co-expression network analysis provided orthogonal confirmation of the single-cell findings at the bulk transcriptomic level. Louvain community detection within the network resolved NPY and NPY1R into a neural-identity community, distinct from the hypoxia-angiogenesis-invasion community comprising VEGFA, ANGPT2, MMP9, LDHA, HK2, and CD274. This segregation into distinct network communities indicates that NPY-pathway and HIF-pathway genes are regulated by distinct transcriptional programs, further arguing against a co-activation model.

VIPER-based TF activity profiling identified HIF1A, EPAS1, STAT3, and RELA among the most active transcription factors in the GBM cohort. The identification of STAT3 is consistent with its known dual role as a HIF-1α transcriptional co-activator and a downstream effector of IL-6/JAK signaling [44,45], corroborating the GSEA enrichment of the IL-6/JAK/STAT3 Hallmark set [NES = 2.53]. The network analysis resolves the NPY axis not as a driver of the hypoxic-immunosuppressive program, but as a parallel, neural-identity-associated transcriptional module whose loss accompanies malignant transformation [46,47].

### 3.6. Prognostic Implications and Therapeutic Considerations

The LASSO–Cox risk model, which integrates expression of NPY-panel genes into a composite score, demonstrated prognostic value independent of IDH mutation status and tumor grade. This is clinically relevant because it suggests the NPY-axis expression profile captures biological information orthogonal to established molecular classifiers [48,49]. The direction of prognostic association—high expression of hypoxia/invasion genes predicting worse outcomes, and high NPY/NPY1R predicting better outcomes—is consistent with the loss-of-NPY model proposed here. Therapeutically, the results suggest two speculative directions warranting further investigation. First, the HIF-2α [EPAS1] inhibitor belzutifan [MK-6482; originally PT2977], currently in clinical trials for solid tumors, could in principle target the hypoxia-driven component, though its relevance to NPY-axis restoration remains untested [50]. Second, rather than NPY receptor antagonism, whether pharmacological restoration or mimicry of NPY signaling could counteract the loss of neuroprotective, anti-inflammatory neuropeptide signaling remains an open question that would require functional validation in preclinical GBM models [8]. These findings support a model in which loss of NPY-axis expression accompanies the transition from neural-like to hypoxia-enriched mesenchymal tumor states; however, the data do not establish direct causal suppression of NPY by hypoxia in GBM and should be interpreted as correlative.

### 3.7. Cross-Platform Considerations

The findings reported here integrate data generated on technically heterogeneous platforms, and the convergent signal across them strengthens biological inference while also warranting interpretive caution. The TCGA-GBM/LGG cohort was profiled by bulk Illumina HiSeq RNA-seq with polyA-selected libraries, which provides high transcript-level dynamic range but averages signal across all cell types within each tumor and may therefore underestimate cell-type-specific NPY expression confined to rare neural-progenitor-like populations. The GEO validation cohorts GSE4290 and GSE50161 were generated on Affymetrix HG-U133 Plus 2.0 microarrays, whose probe-level detection, compressed dynamic range, and gene-coverage limitations partly explain the higher between-study heterogeneity [I^2^ > 75%] observed for several NPY-panel genes in the meta-analysis [Table 3]; accordingly, microarray-derived effect sizes were interpreted alongside, rather than averaged uncritically with, RNA-seq estimates. The single-cell GSE131928 dataset was generated on the 10X Chromium 3′ platform, which yields sparse, dropout-prone counts with 3′ end bias; we mitigated these characteristics through SCTransform normalization, Harmony batch correction, and DoubletFinder filtering, but low-abundance transcripts such as NPY1R remain susceptible to under-detection [<2% of cells expressing in any population], and the absence of NPY ligand–receptor pairs in CellChatDB further constrains direct communication inference. The spatial transcriptomics dataset GSE194329 was acquired on the 10X Visium platform, in which each 55 µm spot captures 1–10 cells of mixed identity; this resolution is well-suited to mapping module-level co-localization [e.g., Hypoxia Core × Glycolysis, r = 0.746] but is too coarse to resolve single-cell NPY signaling events and may explain the weak global NPY–Hypoxia bivariate Moran’s I observed across spots. Together, these platform-specific characteristics suggest that the cross-modality consistency of NPY/NPY1R downregulation reflects a robust biological signal rather than a technical artifact, while the heterogeneity of NPY2R and NPY5R findings is most parsimoniously attributed to platform sensitivity and probe/transcript coverage differences.

## 4. Materials and Methods

### 4.1. Datasets and Specimen Provenance

All analyses were performed on publicly available, de-identified human transcriptomic datasets; no new patient material was collected for this study. [i] TCGA-GBM [*n* = 169 IDH-wildtype GBM surgical resections] and TCGA-LGG [*n* = 510 lower-grade glioma resections] were obtained from the Genomic Data Commons [GDCs] portal; clinical metadata, including age, sex, IDH mutation status, MGMT promoter methylation, and overall survival were retrieved from the cBioPortal PanCancer Atlas. [ii] GTEx v8 normal frontal cortex tissue [*n* = 207 donors] served as the non-tumor reference. [iii] GSE4290 [Sun et al.] provided 180 primary surgical glioma resections [77 GBM, 45 oligodendroglioma, 26 astrocytoma, and 23 non-tumor controls] profiled on Affymetrix HG-U133 Plus 2.0 (Lasobiotech, Suzhou, China) microarrays. [iv] GSE50161 included 130 brain tumor specimens [34 GBM plus other CNS tumors and 13 non-tumor controls] on the same array platform. [v] GSE131928 [Neftel et al.] provided 10X Chromium scRNA-seq of ~20,426 cells from 28 patients IDH-wildtype GBM. [vi] GSE194329 provided 10X Visium spatial transcriptomics from six tissue sections of five adult GBM patients, with annotated tumor core, pseudo-palisading, and infiltrating-edge regions. All tumor specimens were primary surgical resections; recurrent tumors were excluded from survival analyses.

### 4.2. Study Design and Gene Panel Definition

We designed a multi-scale transcriptomic study to characterize the relationship between neuropeptide Y [NPY] signaling and hypoxia-driven programs in GBM [GBM]. The analysis pipeline integrates the following seven sequential layers: [i] bulk RNA-seq differential expression analysis [TCGA], [ii] survival modeling, [iii] pathway enrichment, [iv] external validation and meta-analysis [GEO], [v] single-cell RNA-seq, [vi] tumor microenvironment and cell–cell communication analysis, and [vii] spatial transcriptomics, complemented by transcription factor activity inference and co-expression network construction.

### 4.3. TCGA Bulk RNA-Seq Differential Expression Analysis

Raw RNA-seq count data [STAR–Counts workflow] for TCGA-GBM [*n* = 169] and TCGA-LGG [*n* = 510] were downloaded via TCGAbiolinks [30]. Normal brain reference expression was obtained from GTEx via recount3, specifically the BRAIN project [GENCODE v26]. Ensembl gene identifiers were mapped to HGNC symbols using org.Hs.eg.db. Low-abundance genes were filtered using edgeR::filterByExpr, and library sizes were normalized by the trimmed mean of M-values [TMM] method. The normalized count matrix was transformed using limma::voom [31], and linear models were fitted with trend-robust empirical Bayes moderation. The following four pairwise comparisons were performed: (1) normal brain vs. GBM, (2) LGG vs. GBM, (3) Mesenchymal vs. Proneural GBM, and (4) Mesenchymal vs. Classical GBM. Genes with |log_2_FC| > 0.5 and BH-adjusted *p* < 0.05 were considered significantly differentially expressed.

### 4.4. Survival Analysis

Overall survival data were extracted from TCGA clinical annotations for GBMLGG patients with complete follow-up. Of the combined TCGA-GBM [*n* = 169] and TCGA-LGG [*n* = 510] cohorts [total *n* = 679], 311 patients had complete overall-survival metadata [OS time and event status both non-missing] and passed quality-control criteria [RIN ≥ 6, IDH status annotated, primary tumors only; recurrent and secondary tumors excluded]. All survival modeling was restricted to this analytic cohort *n* = 311 [selected from 679 TCGA-GBMLGG patients with complete overall survival data]. Patients were stratified into high- and low-expression groups at the median expression of each gene. Kaplan–Meier survival curves were compared using the log-rank test with BH correction. Univariate Cox proportional hazards regression was performed for each gene, with hazard ratios, 95% confidence intervals, and concordance indices reported. Multivariate Cox regression adjusted for age, tumor grade, and IDH mutation status. All expression variables were z-score-standardized prior to model fitting. A LASSO-penalized Cox regressFion model was constructed using glmnet [35] with L1 penalty and 10-fold cross-validation. The regularization parameter λ was selected to minimize cross-validation error. The composite risk score was computed as the weighted sum of gene expression values using non-zero LASSO coefficients, and patients were dichotomized at the median risk score into high- and low-risk groups. Time-dependent ROC analysis at 1-, 2-, 3-, and 5-year endpoints was performed using the timeROC package.

### 4.5. Pathway Enrichment Analysis

Gene Set Enrichment Analysis [GSEA] was performed using fgsea [36] on ranked gene lists constructed as sign[log_2_FC] × [−log_10_[adjusted *p*]]. Gene set collections from MSigDB were obtained via msigdbr as follows: Hallmark [50 sets], C2-CGP [chemical and genetic perturbations], and C5-GO: BP [Gene Ontology Biological Process]. GSEA was run with 10,000 permutations, a minimum gene set size of 15, and a maximum size of 500. Normalized enrichment scores [NESs] and BH-adjusted *p*-values were reported. Over-representation analysis [ORA] was performed with clusterProfiler [51]. Significantly differentially expressed NPY-panel genes were used as the foreground set, with all genes tested in the DEA as background. KEGG, Reactome, and GO Biological Process enrichment analyses were performed with BH correction [*p* < 0.05, *q* < 0.2]. GO BP results were simplified using semantic similarity [cutoff = 0.6] to reduce redundancy.

### 4.6. External Validation and Meta-Analysis

Independent validation was performed using the following two microarray datasets from Gene Expression Omnibus: GSE4290 [GBM vs. epilepsy brain] and GSE50161 [GBM vs. normal brain]. Microarray data were log_2_-transformed and quantile-normalized. Probe-to-gene mapping was performed using best-probe collapse based on mean expression per gene. Differential expression analysis was performed for each cohort using the same limma framework as in the TCGA analysis. Random-effect meta-analysis was conducted using metafor [38] with restricted maximum likelihood [REML] estimation and inverse-variance weighting across three cohorts [TCGA-GBMLGG, GSE4290, and GSE50161]. Heterogeneity was quantified by τ^2^, I^2^, and Cochran’s Q test. *p*-values were corrected using BH adjustment. Genes with I^2^ > 75% were flagged as highly heterogeneous.

### 4.7. Single-Cell RNA-Seq Analysis

We reanalyzed single-cell RNA-seq data from GSE131928 [39], comprising tumor cells from adult IDH-wildtype GBM patients profiled on 10X Chromium and Smart-seq2 platforms. Pediatric samples were excluded. Quality control filtering was applied adaptively per sample as follows: cells were retained if mitochondrial content was <25%, ribosomal content was <40%, and feature and unique molecular identifier [UMI] counts fell within ±3 median absolute deviations [MADs] of the per-sample median. Expression values were normalized using SCTransform v2 with regression of mitochondrial percentage and UMI count. Batch effects between platforms were corrected using Harmony [28]. Doublets were identified and removed per sample using DoubletFinder with an expected doublet rate of 0.8% per 1000 cells. Dimensionality reduction was performed with principal component analysis [PCA; 50 components], followed by shared nearest-neighbor graph construction on the Harmony embedding and Louvain clustering. UMAP embeddings were generated for visualization. Cell-type annotation was performed by computing module scores [Seurat::AddModuleScore] for 13 predefined cell-type-specific gene signatures; each cell was assigned to the type with the highest module score.

### 4.8. Tumor Microenvironment and Cell–Cell Communication

Macrophage polarization was assessed by computing M1 and M2 signature scores using AddModuleScore on macrophage-annotated cells. The M1 signature comprised *TNF*, *IL6*, *IL1B*, *CD80*, *NOS2*, *CXCL10*, *HLA-DRA*, *STAT1*, and *CXCL9*. The M2 signature comprised *MRC1*, *CD163*, *ARG1*, *IL10*, *TGFB1*, *CD274*, *CCL22*, *VEGFA*, and *LYVE1*. Cell–cell communication was inferred using CellChat v1.5 [52] with the human CellChatDB ligand–receptor database. Significant interactions [*p* < 0.05] were identified for all cell-type pairs. Interactions were filtered for pathways relevant to the NPY-hypoxia-immune axis: *SPP1*, *PDGF*, *ANGPTL*, *TGFb*, and *VEGF. NPY–NPY1R/NPY2R* ligand–receptor pairs are not represented in CellChatDB; this limitation is addressed in the Discussion.

### 4.9. Spatial Transcriptomics

Spatial gene expression data from six 10X Visium sections [5 GBM patients] were obtained from GSE194329. Quality control removed spots with <200 detected features, <500 UMIs, or >30% mitochondrial content. Expression was normalized using SCTransform with regression of mitochondrial percentage. Dimensionality reduction, clustering, and UMAP visualization were performed using standard Seurat workflows. Spatial regions were annotated by computing module scores for 12 biological cluster signatures based on curated marker gene lists, with each spot assigned to the highest-scoring annotation. The seven NPY-Hypoxia module scores were projected onto tissue coordinates and z-scored within each sample. Differences in module scores across spatial regions were tested using the Kruskal–Wallis test with BH correction. Spatial cell-type deconvolution was performed with SPOTlight [29] via non-negative matrix factorization and non-negative least squares, using the scRNA-seq cell-type annotations [GSE131928] as the reference.

### 4.10. Transcription Factor Activity and Co-Expression Network Analysis

Transcription factor [TF] activity was inferred using VIPER [53] with DoRothEA regulons [40] filtered to confidence levels A, B, and C [minimum 10 target genes per TF]. The input was a log_2_[CPM] expression matrix from the TCGA cohort. TF activity scores were computed per sample. The following fifteen NPY-axis-relevant TFs were tracked: *HIF1A*, *EPAS1*, *SP1*, *STAT3*, *MYC*, *RELA*, *TP53*, *NFE2L2*, *YAP1*, *TEAD1*, *ETS1*, *ARNT*, *TWIST1*, *SNAI1*, and *ZEB1*. A gene–gene co-expression network was constructed from NPY-panel genes and the top 80 co-expressed genes in the TCGA dataset. Edges were retained for gene pairs with |Pearson *r*| ≥ 0.50 and BH-adjusted *p* < 0.05. Louvain community detection was applied to identify network modules. An integrative multi-omic heatmap combined z-scored bulk log_2_FC, meta-analytic significance [−log_10_[FDR]], and VIPER TF activity for the 22 NPY-panel genes.

### 4.11. Composition Correction and Spatial Cross-Correlation

To assess whether bulk differential expression of NPY-axis genes could be attributed to changes in cell-type composition rather than to gene-intrinsic regulation, we performed an analytical composition correction. Cell-type expression profiles for *NPY* and *NPY1R* were derived from the scRNA-seq atlas [GSE131928], and neural lineage fractions were estimated from published single-cell deconvolution studies. The composition-expected log_2_FC was calculated as log_2_[Σ f_GBM × e_i/Σ f_normal × e_i], where f represents cell-type fractions and e represents mean expression per cell type. The residual log_2_FC [observed minus expected] quantifies the proportion of differential expression unexplained by composition shift. To evaluate spatial cross-correlation between module scores while accounting for spatial autocorrelation, we computed bivariate Moran’s I [I_biv] using k = 6 nearest-neighbor spatial weights and 999 permutations. The statistic was calculated as I_biv = [N/Σw_ij] × Σ w_ij × z_x[i] × z_y[j], where z_x and z_y are standardized module scores for NPY Signaling and Hypoxia Core, respectively. Per-sample and pooled statistics were computed. As a positive control, Hypoxia Core × Glycolysis I_biv was computed to confirm the method’s sensitivity to known colocalization.

### 4.12. Software and Reproducibility

All statistical analyses were performed in R (v4.0; R Foundation for Statistical Computing, Vienna, Austria). Key software packages included: limma (v3.58), Seurat (v5.0), CellChat (v1.5), SPOTlight (v1.6), metafor (v4.4), fgsea (v1.28), and glmnet (v4.1). This study is entirely in silico; no physical laboratory equipment was used.

All random seeds were fixed to 42 for reproducibility. Complete analysis scripts are openly available in a public GitHub repository (https://github.com/itika05/NPY-GBM-transcriptomics access on 20 April 2026).

## 5. Limitations

Several limitations should be considered. First, this study is, by design, an integrative in silico investigation; it relies entirely on transcriptomic data and does not capture protein abundance, receptor localization, or in situ signaling activity. The findings reported here should therefore be regarded as hypothesis-generating and require systematic experimental validation along the following axes: [i] qPCR quantification of NPY and NPY1R transcripts in matched GBM and peritumoral brain tissue; [ii] Western blot confirmation of NPY and NPY1R protein levels; [iii] immunohistochemistry on a GBM tissue microarray, stratified by IDH status and transcriptional subtype [proneural, classical, and mesenchymal], to map receptor localization; [iv] functional in vitro assays in established GBM cell lines [U87, U251] and patient-derived glioma stem cells, including NPY1R knockdown and overexpression followed by proliferation, migration, invasion, and clonogenic assays under both normoxic and hypoxic [1% O_2_] conditions; [v] ELISA measurement of secreted NPY in conditioned media; [vi] co-culture of GBM cells with THP-1–derived macrophages to test whether NPY restoration shifts polarization toward an M2 phenotype; and [vii] in vivo orthotopic xenograft studies with NPY1R modulation to assess effects on tumor growth, vascular remodeling, and immune infiltration. Second, both the survival analysis [Table 2] and the prognostic model are based exclusively on TCGA data. As such, the model requires external validation before any clinical interpretation, which was not feasible here due to the lack of follow-up data in GSE4290 and GSE50161. Third, the meta-analysis includes only three cohorts, limiting power to assess heterogeneity. In addition, the composition-correction approach depends on external estimates of cell-type proportions and single-cell reference signatures, which may introduce modeling uncertainty. Fourth, although NPY–NPY1R/NPY2R pairs are not present in CellChatDB, our custom permutation-based ligand–receptor analysis provides only transcript-level support and cannot exclude non-transcriptional or low-abundance signaling mechanisms. Finally, the spatial transcriptomic dataset is relatively small [six sections from five patients], and the weak spatial cross-correlation between NPY and hypoxia programs [pooled Moran’s I = 0.002] suggests that larger spatial datasets will be needed to better resolve niche organization.

## 6. Conclusions

This study presents a comprehensive multiomics characterization of NPY signaling in GBM. Through the integration of bulk transcriptomic meta-analysis, single-cell and spatial RNA sequencing, intercellular communication modeling, and network-level regulatory inference, we establish that the loss of *NPY* expression is a conserved, grade-dependent feature of GBM that is inversely associated with hypoxia-driven, immunosuppressive transcriptional programs. Key conclusions include: [i] NPY and NPY1R are consistently downregulated across TCGA and two independent GEO cohorts, while *NPY2R* upregulation is TCGA-specific and not validated; [ii] at single-cell resolution, NPY-axis and HIF-hypoxia programs are compartmentalized to neural progenitor-like and mesenchymal tumor cells, respectively, rather than co-activated; [iii] intercellular communication in the GBM TME is dominated by SPP1, PDGF, and TGFb signaling rather than NPY; [iv] co-expression network analysis confirms NPY/NPY1R and hypoxia/invasion genes segregate into distinct communities; and [v] the LASSO-Cox risk score integrating NPY-panel gene expression provides independent prognostic value. These findings reposition NPY not as an active driver of the hypoxic-immunosuppressive GBM microenvironment, but as a neural-identity marker whose progressive loss accompanies malignant transformation.

## Figures and Tables

**Table 1:** Differential expression summary for 22 *NPY*-panel genes in the normal brain vs. GBM comparison [TCGA GBMLGG; limma-voom]. Log2FC, average expression [AveExpr], moderated t-statistic, raw *p*-value, Benjamini–Hochberg [BH]-adjusted *p*-value, and direction of change [UP/DOWN] are reported for each gene. Genes with |log2FC| > 0.5 and BH-adjusted *p* < 0.05 are classified as significantly differentially expressed. The functional module assignment for each gene is indicated [*NPY* axis, hypoxia, angiogenesis, metabolism, stemness, invasion, immune suppression].

Gene	log_2_FC	*p*-Value	Adj. *p*
		Normal vs. GBM	
*POU5F1*	5.037	<2.2 × 10^−308^	<2.2 × 10^−308^
*NES*	3.7077	<2.2 × 10^−308^	<2.2 × 10^−308^
*HIF1A*	1.927	<2.2 × 10^−308^	<2.2 × 10^−308^
*MMP2*	4.1653	<2.2 × 10^−308^	<2.2 × 10^−308^
*HK2*	3.7783	<2.2 × 10^−308^	<2.2 × 10^−308^
*MMP9*	4.0507	<2.2 × 10^−308^	<2.2 × 10^−308^
*VEGFA*	2.6616	<2.2 × 10^−308^	<2.2 × 10^−308^
*ANGPT2*	2.8459	<2.2 × 10^−308^	<2.2 × 10^−308^
*LDHA*	1.5412	2.58 × 10^−265^	7.15 × 10^−265^
*SOX2*	1.998	5.72 × 10^−184^	1.27 × 10^−183^
*PROM1*	1.6325	1.57 × 10^−178^	3.44 × 10^−178^
*NPY1R*	−3.187	1.90 × 10^−152^	3.86 × 10^−152^
*CD274*	1.1226	1.60 × 10^−113^	2.89 × 10^−113^
*TGFB1*	0.8655	2.32 × 10^−105^	4.08 × 10^−105^
*NPY5R*	−2.8658	4.81 × 10^−94^	8.12 × 10^−94^
*PDGFRB*	1.0995	1.07 × 10^−82^	1.74 × 10^−82^
*SLC2A1*	0.6433	3.46 × 10^−36^	4.65 × 10^−36^
*NPY2R*	1.6652	7.41 × 10^−34^	9.84 × 10^−34^
*BNIP3*	−0.3164	2.92 × 10^−33^	3.87 × 10^−33^
*ARG1*	0.6612	2.38 × 10^−26^	3.03 × 10^−26^
*EPAS1*	0.4769	6.61 × 10^−18^	8.02 × 10^−18^
*NPY*	−1.4254	5.87 × 10^−14^	6.94 × 10^−14^
		LGG vs. GBM	
*VEGFA*	2.9407	8.18 × 10^−156^	3.46 × 10^−154^
*MMP9*	4.7734	1.32 × 10^−135^	3.77 × 10^−134^
*LDHA*	1.782	5.32 × 10^−123^	1.16 × 10^−121^
*ANGPT2*	2.408	4.23 × 10^−122^	9.03 × 10^−121^
*ARG1*	1.4344	1.09 × 10^−61^	5.28 × 10^−61^
*HK2*	1.2887	5.67 × 10^−56^	2.41 × 10^−55^
*SOX2*	−0.9827	3.80 × 10^−46^	1.30 × 10^−45^
*PDGFRB*	0.7508	1.41 × 10^−44^	4.67 × 10^−44^
*POU5F1*	0.8931	1.32 × 10^−37^	3.75 × 10^−37^
*NPY*	−1.9326	8.07 × 10^−35^	2.16 × 10^−34^
*CD274*	1.0102	1.57 × 10^−32^	3.97 × 10^−32^
*NPY1R*	−1.3709	6.41 × 10^−28^	1.47 × 10^−27^
*MMP2*	0.7922	3.31 × 10^−22^	6.65 × 10^−22^
*NPY2R*	1.5102	6.62 × 10^−19^	1.23 × 10^−18^
*PROM1*	0.7598	2.27 × 10^−16^	3.96 × 10^−16^
*NPY5R*	−1.0965	1.16 × 10^−13^	1.90 × 10^−13^
*EPAS1*	−0.38	4.38 × 10^−13^	7.07 × 10^−13^
*SLC2A1*	0.324	1.02 × 10^−10^	1.55 × 10^−10^
*BNIP3*	−0.2639	4.77 × 10^−8^	6.73 × 10^−8^
*HIF1A*	0.1598	9.00 × 10^−4^	0.0011
*TGFB1*	−0.1823	0.0106	0.0123
*NES*	0.0654	0.3773	0.3967

**Table 2:** Survival analysis summary for 22 NPY-panel genes in the TCGA GBMLGG cohort [*n* = 311 patients with complete overall survival [OS] data]. For each gene: univariate Cox PH regression HR, 95% confidence interval [CI], Wald *p*-value, BH-adjusted *p*-value, and C-index; KM log-rank *p*-value and BH-adjusted *p*-value; multivariate Cox PH regression HR and *p*-value [adjusted for age, grade, and IDH mutation status]; LASSO–Cox coefficient and selection status. Expression values were z-score-standardized before all model fitting.

*Gene*	Module	HR [uni]	95% CI	*p* Adj [uni]	KM Log-Rank *p*	HR [Multi]	LASSO Coef	Signature
*MMP2*	Invasion	1.1886	1.061–1.331	0.0403	0.1318	1.1996	0.1582	Yes
*MMP9*	Invasion	1.1962	1.060–1.350	0.0403	0.0016	1.176	0.1365	Yes
*CD274*	Immune Supp.	1.1365	1.012–1.277	0.2278	0.3525	1.2081	0.1598	Yes
*LDHA*	Metabolism	1.0995	0.986–1.226	0.4766	0.2336	0.9635	-	No
*PROM1*	Stemness	1.0962	0.977–1.230	0.5243	0.1006	1.0803	0.0909	Yes
*NPY2R*	NPY Axis	1.0745	0.967–1.194	0.5566	0.1146	1.0376	0.0665	Yes
*HIF1A*	Hypoxia	0.9347	0.838–1.043	0.5566	0.0240	0.9107	−0.079	Yes
*NPY*	NPY Axis	1.0698	0.958–1.194	0.5566	0.3974	1.0699	0.0143	Yes
*SOX2*	Stemness	0.9546	0.882–1.033	0.5566	0.0013	1.0475	−0.0119	Yes
*PDGFRB*	Angiogenesis	1.0719	0.952–1.208	0.5566	0.5410	0.9575	-	No
*ARG1*	Immune Supp.	0.9407	0.841–1.052	0.5566	0.4820	0.9382	−0.0547	Yes
*HK2*	Metabolism	1.0569	0.951–1.174	0.5566	0.6579	1.0846	0.0329	Yes
*TGFB1*	Immune Supp.	1.0581	0.944–1.186	0.5625	0.5031	0.9053	−0.0517	Yes
*SLC2A1*	Metabolism	0.9533	0.849–1.071	0.6567	0.2864	0.9633	−0.0537	Yes
*NPY5R*	NPY Axis	1.0432	0.935–1.164	0.6567	0.3801	1.0551	-	No
*ANGPT2*	Angiogenesis	1.0388	0.927–1.164	0.7068	0.5412	1.0264	0.0067	Yes
*POU5F1*	Stemness	1.0257	0.917–1.148	0.7758	0.5925	0.9973	0.0107	Yes
*NPY1R*	NPY Axis	1.0248	0.918–1.144	0.7758	0.8650	0.9242	-	No
*BNIP3*	Metabolism	0.9772	0.876–1.090	0.7758	0.8240	0.9447	−0.0112	Yes
*VEGFA*	Angiogenesis	1.0213	0.916–1.139	0.7758	0.6568	0.9528	-	No
*NES*	Stemness	0.9842	0.888–1.090	0.7960	0.3990	0.8485	−0.0919	Yes
*EPAS1*	Hypoxia	1.0074	0.908–1.118	0.8897	0.3684	0.9676	−0.019	Yes

**Table 3:** Random-effect meta-analysis results for 22 NPY-panel genes across three independent cohorts [TCGA-GBMLGG, GSE4290, and GSE50161]. Pooled log_2_FC estimate, SE, z-statistic, *p*-value, BH-adjusted *p*-value, τ^2^ [between-study variance], *I*^2^ [heterogeneity], and Cochran’s Q test *p*-value are reported for each gene. Genes with *I*^2^ > 75% are flagged as highly heterogeneous.

Gene	Estimate [logFC]	95% CI	Adj. *p*	I^2^ [%]	Direction	Significant
*NPY1R*	−3.0484	−3.415 to −2.682	2.02 × 10^−58^	24.0	DOWN	TRUE
*NES*	3.2078	2.368 to 4.048	7.49 × 10^−13^	92.5	UP	TRUE
*SOX2*	1.5431	1.061 to 2.025	2.39 × 10^−9^	91.8	UP	TRUE
*NPY*	−2.0243	−2.960 to −1.089	9.76 × 10^−5^	84.8	DOWN	TRUE
*BNIP3*	−0.432	−0.632 to −0.232	9.76 × 10^−5^	62.4	DOWN	TRUE
*PDGFRB*	0.7896	0.378 to 1.202	0.0006	84.2	UP	TRUE
*VEGFA*	1.946	0.899 to 2.993	0.0008	92.8	UP	TRUE
*EPAS1*	0.376	0.168 to 0.584	0.001	48.6	UP	TRUE
*ANGPT2*	1.9421	0.778 to 3.106	0.0025	94.5	UP	TRUE
*MMP2*	2.7684	1.045 to 4.492	0.0034	97.7	UP	TRUE
*HIF1A*	1.1458	0.354 to 1.938	0.0088	98.4	UP	TRUE
*HK2*	2.3873	0.602 to 4.172	0.0142	97.9	UP	TRUE
*MMP9*	2.6281	0.671 to 4.585	0.0142	97.6	UP	TRUE
*TGFB1*	1.1965	0.288 to 2.105	0.0147	95.6	UP	TRUE
*NPY5R*	−1.4429	−2.906 to 0.020	0.0745	97.9	DOWN	FALSE
*PROM1*	1.5492	−0.064 to 3.162	0.0772	97.0	UP	FALSE
*CD274*	0.7531	−0.039 to 1.545	0.0772	96.4	UP	FALSE
*LDHA*	0.6599	−0.315 to 1.635	0.2152	97.9	UP	FALSE
*NPY2R*	0.5604	−0.630 to 1.750	0.3935	96.5	UP	FALSE
*SLC2A1*	0.1041	−0.509 to 0.717	0.7763	95.9	UP	FALSE
*ARG1*	−0.0623	−0.871 to 0.747	0.88	94.3	DOWN	FALSE

**Table 4:** Mean NPY–Hypoxia module scores per cell type from scRNA-seq analysis [GSE131928; ***n*** = 20,426 cells]. Seven functional module scores [NPY axis, HIF-Hypoxia, angiogenesis, glycolysis, stemness, immune suppression, and invasion] are shown as mean values across 13 annotated cell populations. Positive values indicate above-background module activation; negative values indicate below-background activity relative to a random gene-set control.

Cell Type	NPY Axis	Hypoxia	Angiogenesis	Glycolysis	Stemness	Immune Supp.	Invasion
Endothelial	−0.0188	0.0176	0.0661	0.2034	−0.1016	−0.0799	−0.1248
GSC	0.0029	−0.0203	0.0051	0.2358	0.4869	−0.0948	−0.0794
Macrophage	−0.0052	0.0275	0.0051	0.1933	−0.3321	0.2108	0.0181
NK_cell	−0.0141	0.1452	−0.0318	0.2873	−0.1161	−0.0048	−0.1186
Oligodendro	−0.0189	−0.2491	−0.0366	−0.1469	−0.2055	−0.0557	−0.1361
Pericyte	−0.007	0.1114	0.0171	0.2665	−0.0705	−0.0888	−0.1291
T_cell	−0.0128	−0.0021	−0.0338	0.1923	−0.258	0.1054	0.0324
Tumor_AC	0.0058	−0.0181	−0.01	0.243	−0.0311	−0.0799	0.0053
Tumor_MES1	−0.0049	0.1489	0.0579	0.6367	−0.1169	−0.0157	0.1744
Tumor_MES2	−0.0005	0.0709	0.0295	0.4669	0.0282	−0.0151	0.0447
Tumor_NPC1	0.0085	−0.2264	−0.0443	−0.1318	−0.0878	−0.0971	−0.1993
Tumor_NPC2	0.0121	−0.1005	−0.0641	0.0581	0.0659	−0.0849	−0.1633
Tumor_OPC	0.0123	−0.1183	−0.0609	0.0636	0.072	−0.0998	−0.1267

## Data Availability

No new data were created in this study; all analyses used publicly available datasets. TCGA-GBM and TCGA-LGG RNA-seq and clinical data were obtained from the GDC Data Portal (https://portal.gdc.cancer.gov/, 20 April 2026). GTEx normal brain expression data were obtained from the GTEx Portal (https://gtexportal.org/, 20 April 2026). The microarray, single-cell, and spatial transcriptomic datasets are available from the Gene Expression Omnibus under accession numbers GSE4290, GSE50161, GSE131928, and GSE194329 (https://www.ncbi.nlm.nih.gov/geo/, 20 April 2026). All analysis code used to generate the results, tables, and figures in this study is openly available in a public GitHub repository (https://github.com/itika05/NPY-GBM-transcriptomics, 20 April 2026).

## Supplementary Materials

The following supporting information can be downloaded at https://www.mdpi.com/article/10.3390/ijms27136068/s1.

## Abbreviations

The following abbreviations are used in this manuscript:

ANGPT2	Angiopoietin 2
ARG1	Arginase 1
AUC	Area Under the Curve
BH	Benjamini–Hochberg
BNIP3	BCL2 Interacting Protein 3
CD274	Programmed Death-Ligand 1 [PD-L1]
CI	Confidence Interval
CNS	Central Nervous System
DEA	Differential Expression Analysis
EPAS1	Endothelial PAS Domain Protein 1
ES	Enrichment Score
FDR	False Discovery Rate
GBM	Glioblastoma
GEO	Gene Expression Omnibus
GO	Gene Ontology
GSEA	Gene Set Enrichment Analysis
GTEx	Genotype-Tissue Expression
H&E	Hematoxylin and Eosin
HIF	Hypoxia-Inducible Factor
HIF1A	Hypoxia-Inducible Factor 1 Alpha
HK2	Hexokinase 2
IDH	Isocitrate Dehydrogenase
KEGGs	Kyoto Encyclopedia of Genes and Genomes
LASSO	Least Absolute Shrinkage and Selection Operator
LDHA	Lactate Dehydrogenase A
LGG	Lower-Grade Glioma
MAD	Median Absolute Deviation
MAPK	Mitogen-Activated Protein Kinase
MMP2	Matrix Metallopeptidase 2
MMP9	Matrix Metallopeptidase 9
NMF	Non-Negative Matrix Factorization
NNLSs	Non-Negative Least Squares
NPY	Neuropeptide Y
NPY1R	Neuropeptide Y Receptor Y1
NPY2R	Neuropeptide Y Receptor Y2
NPY5R	Neuropeptide Y Receptor Y5
ORA	Over-Representation Analysis
OS	Overall Survival
PCA	Principal Component Analysis
PDGFRB	Platelet-Derived Growth Factor Receptor Beta
PD-L1	Programmed Death-Ligand 1
PI3K	Phosphoinositide 3-Kinase
POU5F1	POU Class 5 Homeobox 1
PROM1	Prominin 1
QC	Quality Control
REML	Restricted Maximum Likelihood
ROC	Receiver Operating Characteristic
SLC2A1	Solute Carrier Family 2 Member 1
SOX2	SRY-Box Transcription Factor 2
TAM	Tumor-Associated Macrophage
TAMs	Tumor-Associated Macrophages
TCGA	The Cancer Genome Atlas
TF	Transcription Factor
TGFB1	Transforming Growth Factor Beta 1
TME	Tumor Microenvironment
UMAP	Uniform Manifold Approximation and Projection
UMI	Unique Molecular Identifier
VEGFA	Vascular Endothelial Growth Factor A
WHO	World Health Organization
