# Supplementary material for: Loss of Neuropeptide Y Signaling Accompanies the Neural-to-Mesenchymal Transcriptional Transition in Glioblastoma: A Multi-Scale Transcriptomic Analysis
Source: Int J Mol Sci. 2026 Jul 6;27(13):6068. doi: 10.3390/ijms27136068 (PMC13361764; doi:10.3390/ijms27136068)
Supplement: Supplementary file 1 [file ijms-27-06068-s001.zip › Supplementary_Figures_FA_20042026.pdf]

International Journal of
Molecular Sciences

Article

Loss of Neuropeptide Y Signaling Accompanies the Neural-to-Mesenchymal Transcriptional Transition in Glioblastoma: A Multi-Scale Transcriptomic Analysis

Fareeha Arshad[1], Arshiya Akbar[1], Mohammed Imran Khan[2], Bushra Rasheed[1], Adnan Hussain[2], Fahad Ali Alghamdi[2], Faisal Abdulhameed Farrash[3], Edwin N. Aroke[4], Khalid Walid Freij[4], itika Arora[1*] and Ahmed Yaqinuddin[1,*]

1 Alfaisal University, College of Medicine, Riyadh 11533, Saudi Arabia.
farshad@alfaisal.edu
arshiyaakbar2019@gmail.com; ;brasheed01@alfaisal.edu; iarora@alfaisal.edu;
ayaqinuddin@alfaisal.edu

2 King Faisal Specialist Hospital and Research Center, Jeddah, Saudi Arabia mikhan@kfshrc.edu.sa; hadnan@kfshrc.edu.sa; fgalghamdi@kfshrc.edu.sa

3 King Faisal Specialist Hospital and Research Center, Riyadh, Saudi Arabia. ffarrash@kfshrc.edu.sa

4 Department of Acute, Chronic & Continuing Care, School of Nursing, University of Alabama at Birmingham, Birmingham, AL 35294, USA; earoke@uab.edu (E.N.A.); kfreij95@uab.edu (K.W.F.)

* Correspondence: IA: iarora@alfaisal.edu and AY:ayaqinuddin@alfaisal.edu

Tel.:+966535050381

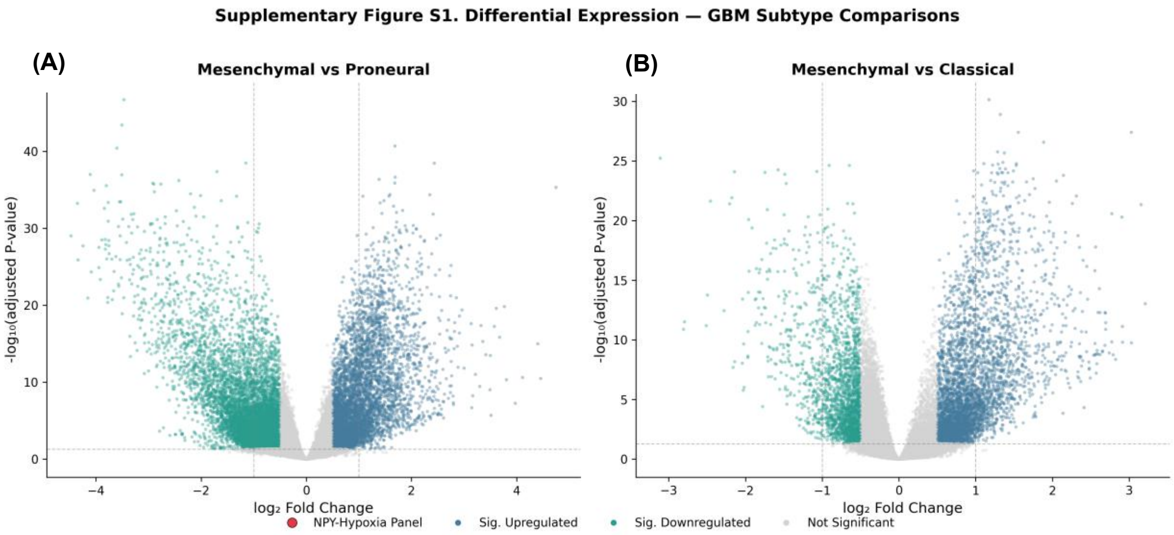

Supplementary Figure S1. Subtype-stratified volcano plots of 22 NPY-panel genes. **(A)** Mesenchymal vs Proneural GBM comparison. **(B)** Mesenchymal vs Classical GBM comparison. NPY-Hypoxia panel genes are highlighted in red. Color coding and significance thresholds (|$\log_2$FC| > 0.5, BH-adjusted P < 0.05) as in Figure 1A. Data derived from TCGA GBMLGG (limma-voom; n = 152 GBM). Gene-level statistics are provided in Supplementary Tables S3 and S4.

International Journal of
Molecular Sciences

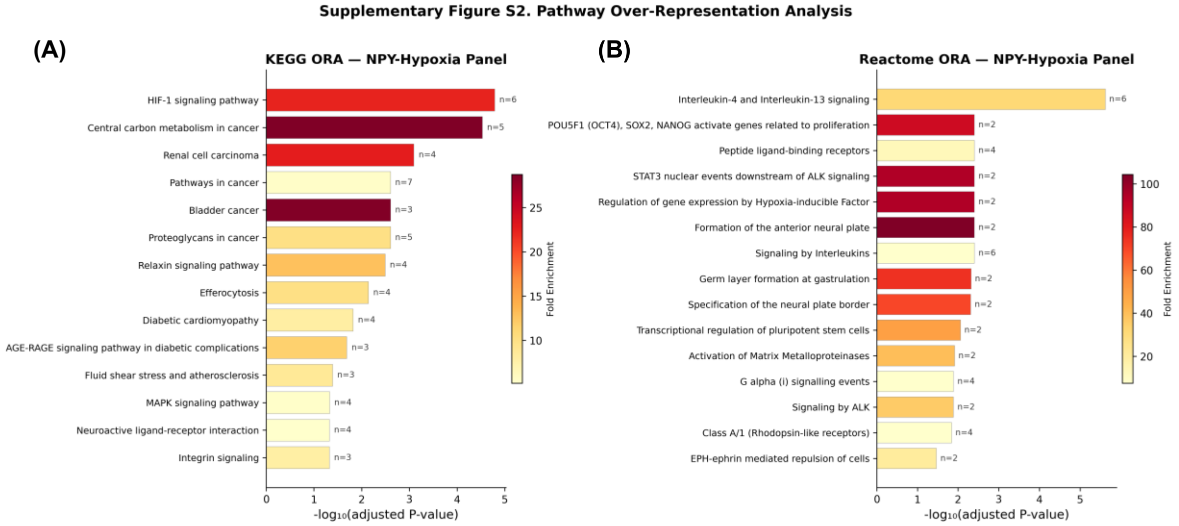

Supplementary Figure S2. Pathway over-representation analysis (ORA) of the 22-gene NPY-Hypoxia panel. **(A)** KEGG pathway ORA showing the top significantly enriched pathways ranked by $-\log_{10}$(adjusted P-value), with bar color representing fold enrichment and gene counts annotated per bar. HIF-1 signaling pathway was the most significant hit. **(B)** Reactome pathway ORA with identical ranking. Interleukin-4 and Interleukin-13 signaling was the top Reactome hit. All pathways shown have BH-adjusted $P < 0.05$. Extends Figure 3: provides granular KEGG and Reactome pathway resolution beyond the GSEA Hallmark analysis shown in the main text.

International Journal of
Molecular Sciences

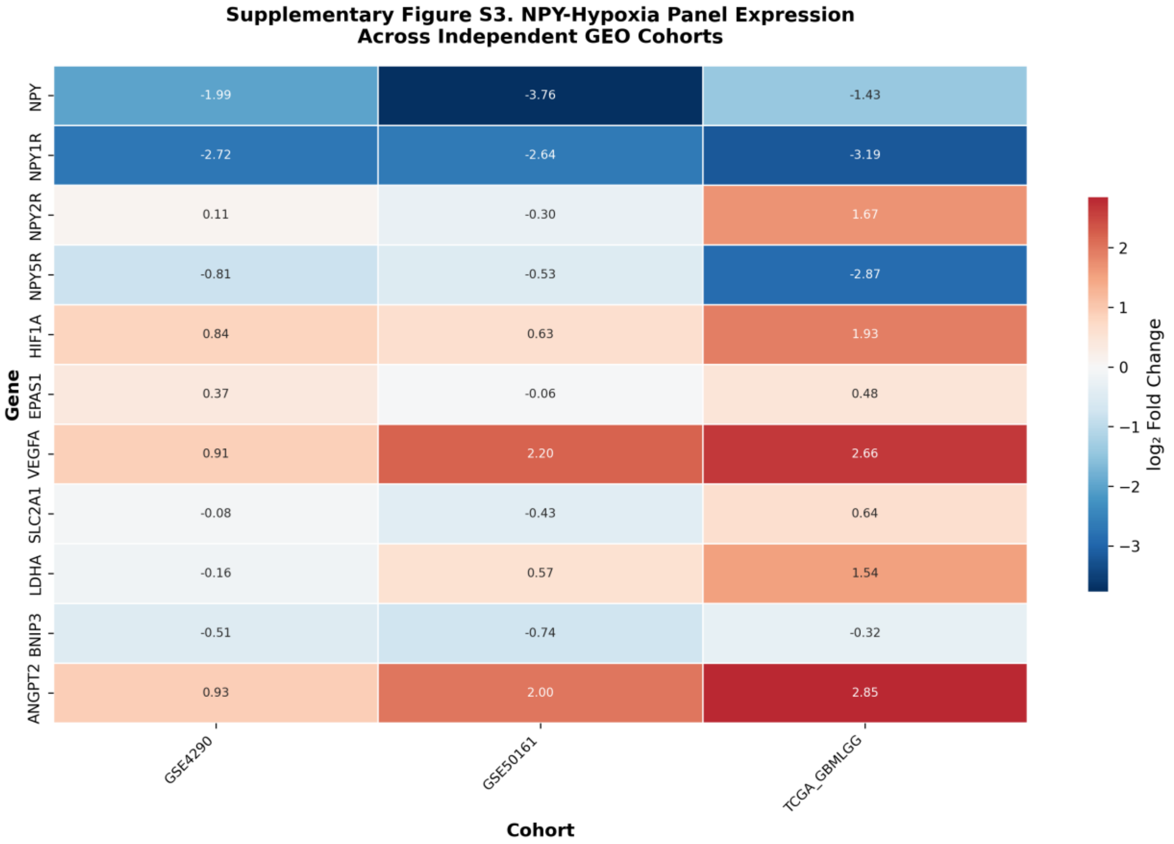

Supplementary Figure S3. NPY-Hypoxia panel expression consistency across independent GEO validation cohorts. Heatmap of $\log_2$ fold change (tumor vs non-tumor) for 12 NPY-panel genes across three cohorts: GSE4290, GSE50161, and TCGA-GBMLGG. Red indicates upregulation in tumor; blue indicates downregulation. Consistent cross-cohort upregulation is evident for VEGFA, ANGPT2, LDHA, and HIF1A, while NPY and its receptors (NPY1R, NPY5R) show consistent downregulation. Extends Figure 4: provides per-cohort detail complementing the pooled random-effects meta-analysis forest plot in the main text.

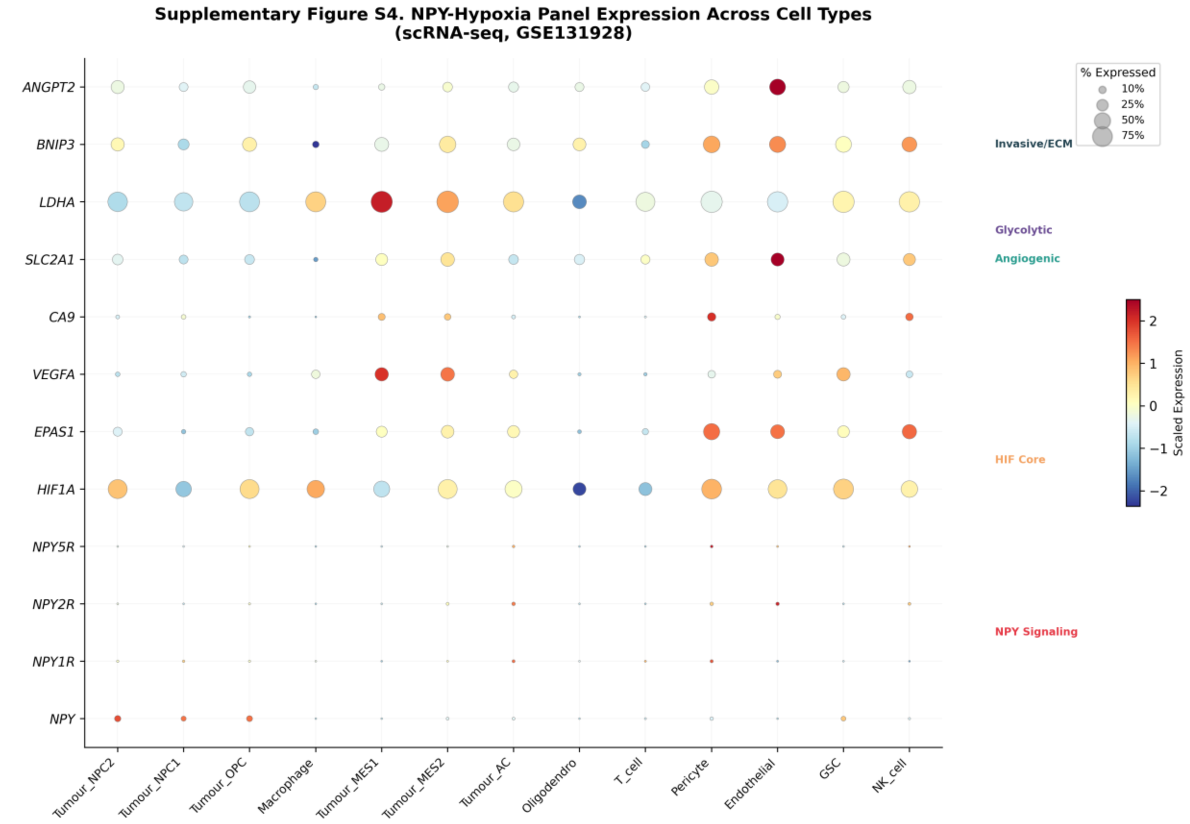

Supplementary Figure S4. Cell-type-resolved expression of the NPY-Hypoxia panel in single-cell RNA-seq data (GSE131928; n = 20,426 cells). Dot plot showing average scaled expression (color) and percentage of cells expressing each gene (dot size) across 13 annotated cell types. Genes are grouped by functional module (NPY Signaling, HIF Core, Angiogenic, Glycolytic, Invasive/ECM). NPY and its receptors show sparse expression, predominantly in neural progenitor-like tumor subtypes (NPC1, NPC2), while HIF1A and LDHA are broadly expressed across mesenchymal subtypes and macrophages.

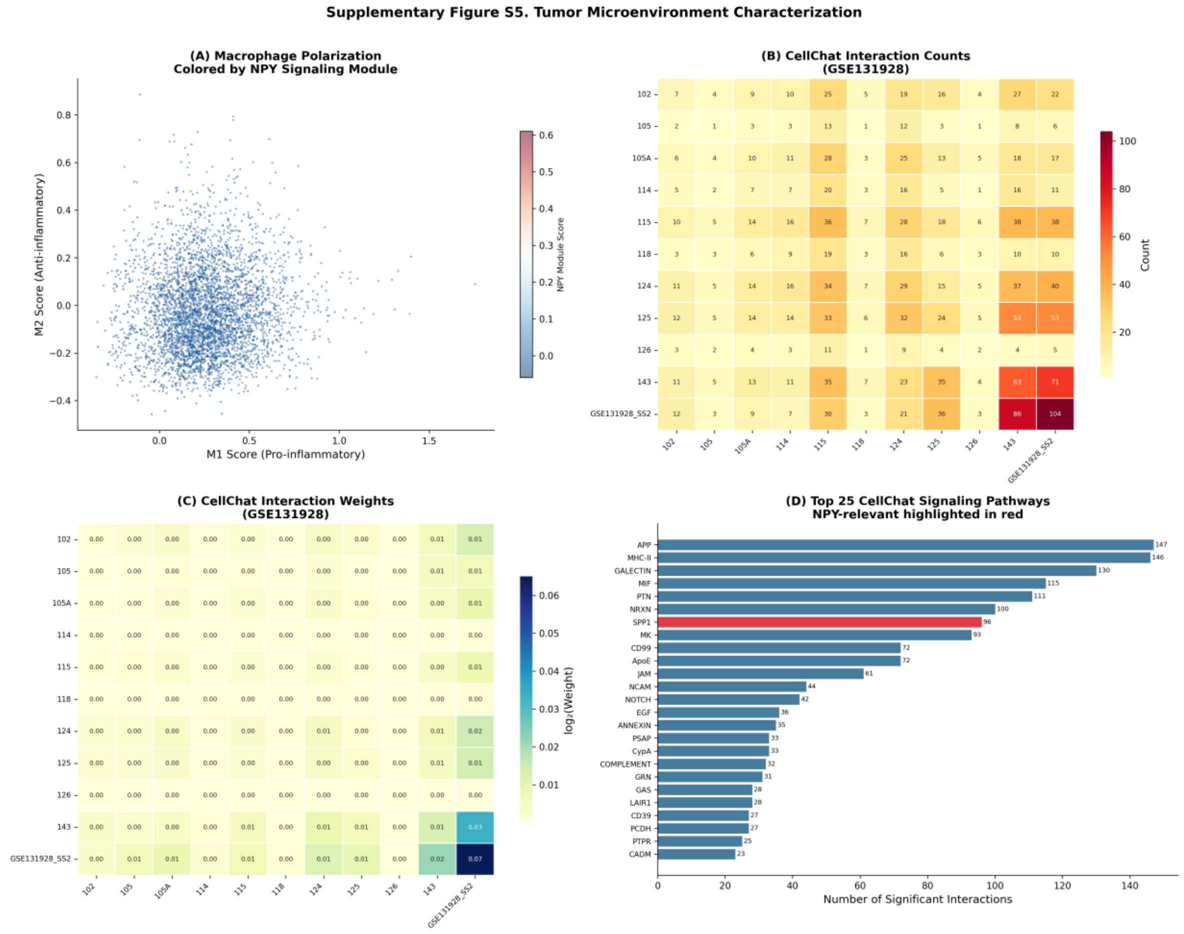

Supplementary Figure S5. Tumor microenvironment characterization and intercellular communication analysis (GSE131928). **(A)** Macrophage M1 (pro-inflammatory) vs M2 (anti-inflammatory) polarization scatter plot, colored by NPY Signaling Module score, showing the heterogeneous distribution of TAM polarization states. **(B)** CellChat interaction count heatmap across all cell-type pairs, revealing dominant sender–receiver relationships. **(C)** CellChat interaction weight (strength) heatmap across cell-type pairs**. (D)** Top 25 CellChat signaling pathways ranked by number of significant interactions, with NPY-relevant pathways highlighted in red (including SPP1, VEGF, PDGF, TGFb, and ANGPTL).

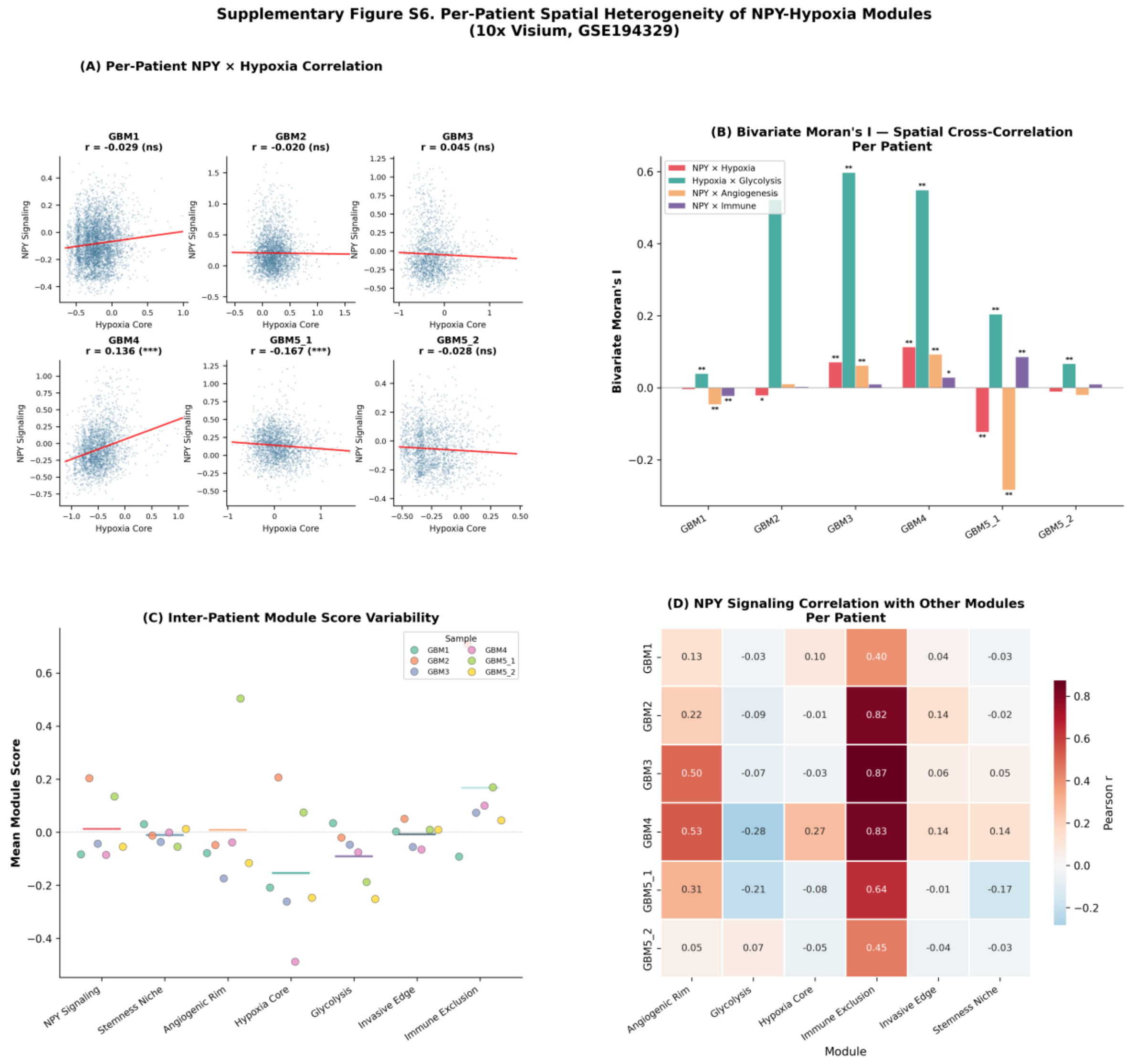

Supplementary Figure S6. Per-patient spatial heterogeneity analysis of NPY-Hypoxia modules (10x Visium, GSE194329; 6 sections, 5 patients). **(A)** Per-patient scatter plots of NPY Signaling vs Hypoxia Core module z-scores with Pearson correlation coefficients (r) and significance annotations. **(B)** Bivariate Moran's I spatial cross-correlation statistics for four module pairs (NPY × Hypoxia, Hypoxia × Glycolysis, NPY × Angiogenesis, NPY × Immune) per patient, revealing patient-specific spatial coupling patterns. **(C)** Inter-patient module score variability dot plot showing mean module activity per patient across seven functional modules. **(D)** NPY Signaling correlation heatmap with all other modules, stratified by patient.

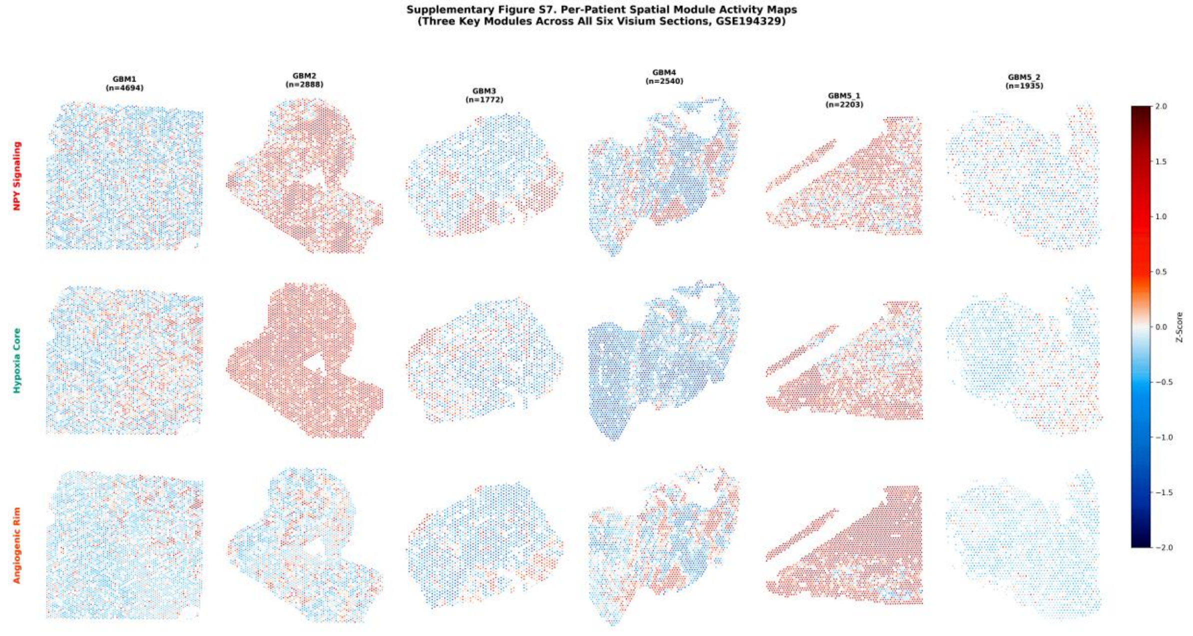

Supplementary Figure S7. Per-patient spatial module activity maps across all Visium sections (GSE194329; 6 sections, 5 patients). Three key modules—NPY Signaling, Hypoxia Core, and Angiogenic Rim—are projected onto tissue coordinates for each patient section. Each row represents one module and each column represents one patient section. Color scale represents z-scored module activity. NPY Signaling shows focal enrichment in neural progenitor-like regions, while Hypoxia Core marks the necrotic core, and Angiogenic Rim localizes to the tumor periphery.

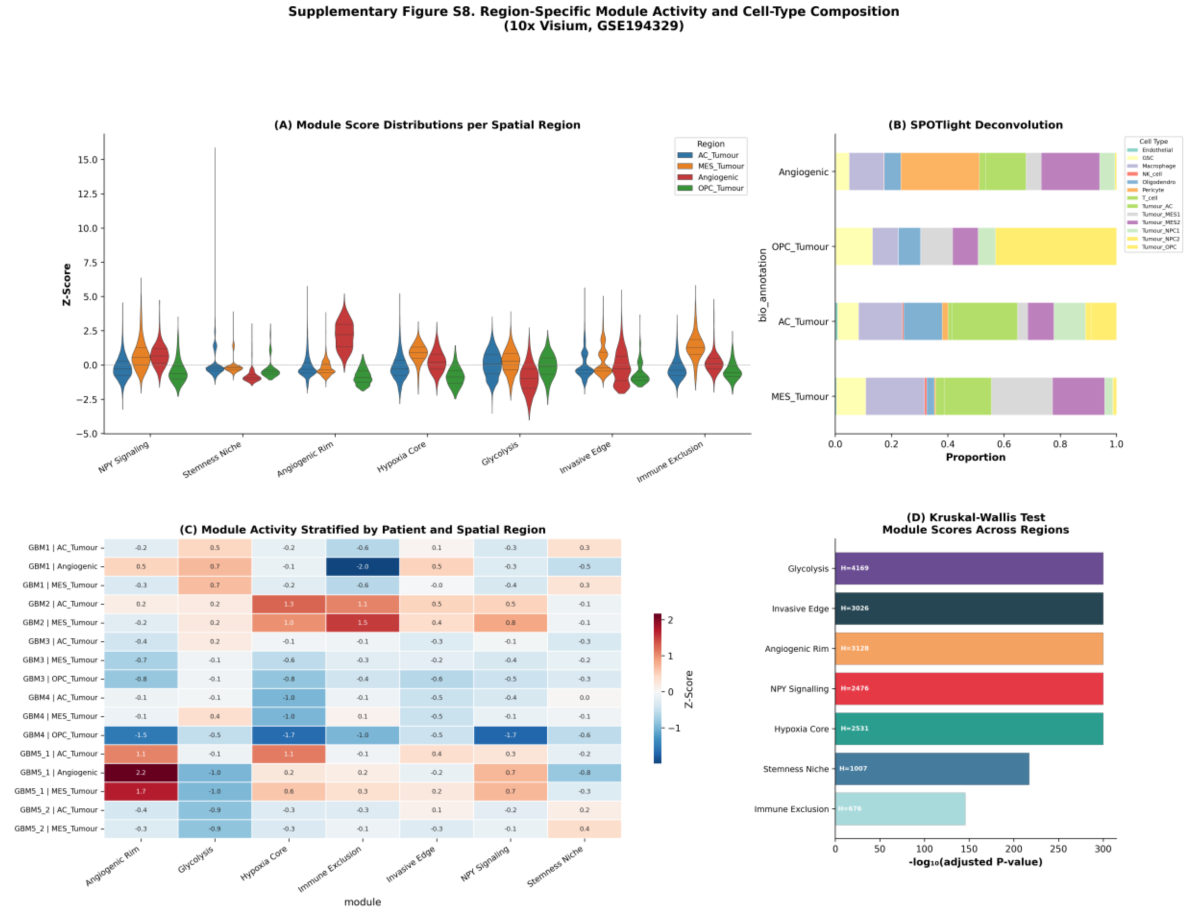

International Journal of
Molecular Sciences

Supplementary Figure S8. Region-specific module activity and cell-type composition in spatial transcriptomics (GSE194329). **(A)** Violin plots of module z-score distributions per annotated spatial region (NPY Signaling, Stemness Niche, Angiogenic Rim, Invasive Edge, Glycolysis, Hypoxia Core, Immune Exclusion) across five zones, showing regional enrichment patterns. **(B)** SPOTlight cell-type deconvolution: region × cell-type proportion stacked bar chart showing inferred cellular composition across four annotated zones. **(C)** Module activity heatmap stratified by both patient sample and spatial region, revealing patient-specific regional patterns. **(D)** Kruskal-Wallis test results for module score differences across spatial regions, showing $-\log_{10}$(adjusted P-value) per module; significant modules are indicated.

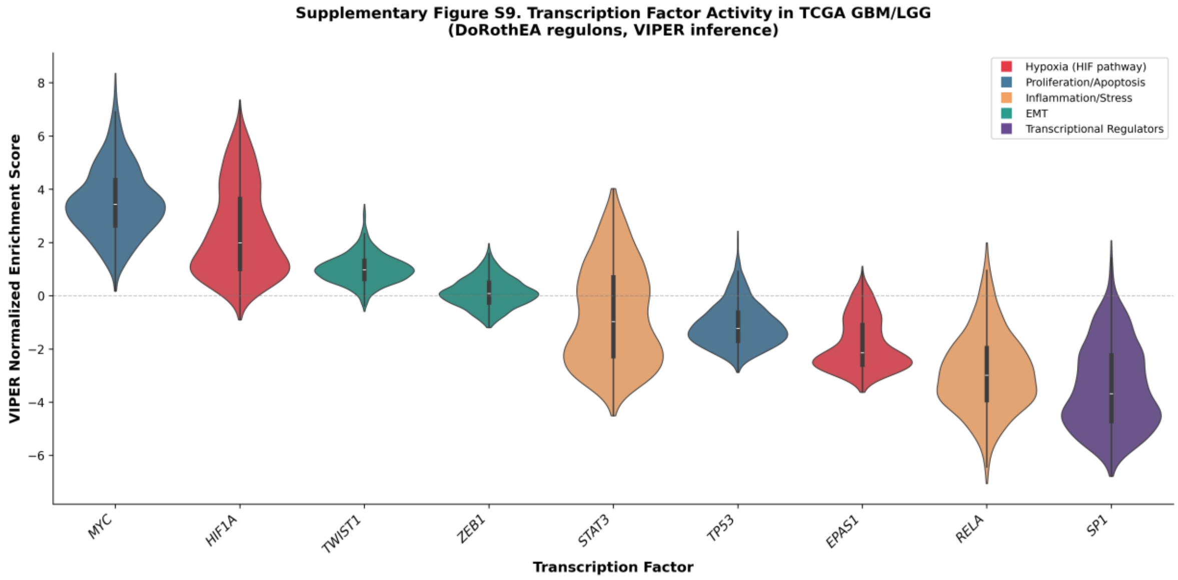

Supplementary Figure S9. Transcription factor activity inference in TCGA GBM/LGG. Violin plots of VIPER-inferred normalized enrichment scores (NES) for 9 representative NPY-axis-relevant transcription factors out of the 15 tracked in the main analysis (MYC, HIF1A, TWIST1, ZEB1, STAT3, TP53, EPAS1, RELA, and SP1), colored by functional category: Hypoxia/HIF pathway (red), Proliferation/Apoptosis (blue), Inflammation/Stress (orange), EMT (teal), and Transcriptional Regulators (purple). HIF1A and MYC show the highest positive activity, while SP1 and RELA show predominantly negative scores. Extends Figure 8 by providing individual TF activity distributions that complement the co-expression network and multi-omic integration matrix in the main text. The remaining 6 tracked TFs (NFE2L2, YAP1, TEAD1, ETS1, ARNT, SNAI1) are included in the co-expression network analysis shown in Figure 8.
